# The Impact of Viral Infection on the Chemistries of the Earth’s Most Abundant Photosynthesizes: Metabolically Talented Aquatic Cyanobacteria

**DOI:** 10.3390/biom13081218

**Published:** 2023-08-04

**Authors:** Yunpeng Wang, Scarlet Ferrinho, Helen Connaris, Rebecca J. M. Goss

**Affiliations:** 1 School of Chemistry, University of St Andrews, North Haugh, St Andrews KY16 9AJ, UK; sf71@st-andrews.ac.uk (S.F.); hc6@st-andrews.ac.uk (H.C.); 2 Biomedical Sciences Research Complex, University of St Andrews, North Haugh, St Andrews KY16 9SX, UK

**Keywords:** cyanophages, cyanobacterial, AMGs, photosynthesis, central carbon metabolism, phosphate metabolism, methylation, regulatory factor

## Abstract

Cyanobacteria are the most abundant photosynthesizers on earth, and as such, they play a central role in marine metabolite generation, ocean nutrient cycling, and the control of planetary oxygen generation. Cyanobacteriophage infection exerts control on all of these critical processes of the planet, with the phage-ported homologs of genes linked to photosynthesis, catabolism, and secondary metabolism (marine metabolite generation). Here, we analyze the 153 fully sequenced cyanophages from the National Center for Biotechnology Information (NCBI) database and the 45 auxiliary metabolic genes (AMGs) that they deliver into their hosts. Most of these AMGs are homologs of those found within cyanobacteria and play a key role in cyanobacterial metabolism-encoding proteins involved in photosynthesis, central carbon metabolism, phosphate metabolism, methylation, and cellular regulation. A greater understanding of cyanobacteriophage infection will pave the way to a better understanding of carbon fixation and nutrient cycling, as well as provide new tools for synthetic biology and alternative approaches for the use of cyanobacteria in biotechnology and sustainable manufacturing.

## 1. Introduction

**Scheme 1:**
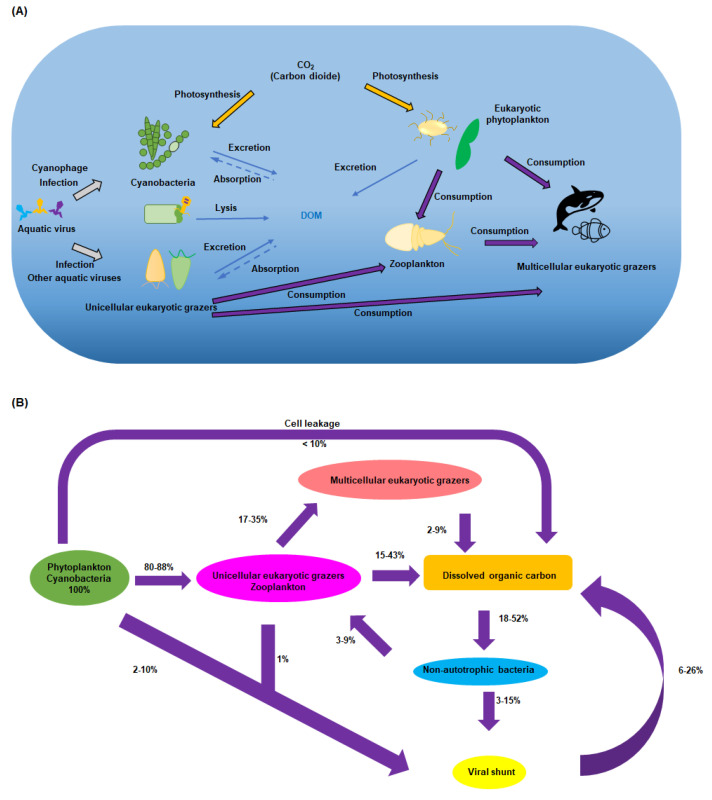
A simplified summary of the central metabolic role that aquatic viruses play. (**A**) Cyanophages play a key role in the mortality and life cycle of photosynthetic bacteria, while other aquatic viruses impact unicellular eukaryotic grazers. These organisms, at the bottom of the food chain, in turn impact all organisms in the ocean and are thus central to carbon flux. (**B**) The steady-state model of organic carbon. (Adapted from reference [7]). All values are expressed in terms of the flux of photosynthetically fixed carbon (100%) The assumption is made that all organic carbon, present in pelagic waters, is readily accessible to bacteria and is eventually respired, with minimal loss through export.

More than half of the oxygen in the atmosphere is generated by oxygenic photosynthetic bacteria, known as cyanobacteria [1]. These bacteria, whose biomass exceeds the total mass of zooplankton and fish, play a central role in carbon capture, the release of dissolved organic carbon (DOC), and nutrient cycling (Scheme 1A). It is becoming increasingly apparent that cyanobacterial metabolic pathways, and nutrient cycling itself, are largely governed by the bacteriophages that infect them. With approximately 10 million viruses in every milliliter of seawater [2] and representing more than 94% of nucleic acid entities in the oceans [3], oceanic viruses are ~10-fold more abundant than marine bacteria and considerably outnumber other marine organisms, such as phytoplankton, zooplankton, and organisms at higher trophic levels [1,4].

Based on published data regarding labile DOC release mediated by microbial and zooplankton activities, the partitioning of inputs among these sources suggests (even though much remains to be understood about the exact nature of the chemistry of these short-lived species) that about 20% of labile DOC is released through excretion and death processes of heterotrophic organisms, approximately 40% originates directly from phytoplankton (including cyanobacteria) through extracellular release during photosynthesis, and around 40% is released through phytoplankton death processes, including senescence, sloppy feeding, and viral and fungal lysis [5,6]. The viral shunt, through the incorporation of carbon in new viruses and the release of carbon during cell lysis, represents a considerable pathway through which DOC is mobilized and redistributed within ecosystems. It is estimated that as much as one-quarter of DOC undergoes the viral shunt process (Scheme 1B) [7]. This highlights the important role that viruses play in the dynamics of carbon cycling, shaping carbon availability and utilization by other organisms in the ecosystem. A greater understanding of the viral shunt process and the chemistries encoded by viruses will provide insights into the intricate connections between viruses, host cells, and the carbon cycle, with implications for ecosystem functioning and biogeochemical processes [7].

Infection of a cyanobacterium by a virus known as a “cyanophage” or “cyanobacteriophage” can be persistent or lethal, ultimately resulting in host lysis and the release of the progeny phage. In both types of infection, host and phage may exchange genetic material. This results in cyanophage often encoding metabolism-related genes, analogous to cyanobacterial genes. These genes, known as auxiliary metabolic genes (AMGs), play a key role in host metabolism following infection. Here, we analyze AMGs from 153 publicly accessible cyanophage genomes [8,9,10,11] (see Table 1) with the aim of gaining greater insight into the ways in which cyanophages manipulate host metabolism. Cyanophages exhibit a broad distribution across diverse aquatic environments worldwide (Table 1). They are prevalent in both freshwater and marine ecosystems, encompassing lakes, rivers, ponds, oceans, and estuaries. The geographic range of cyanophages can vary, influenced by factors such as local environmental conditions, host cyanobacteria species, and the presence of suitable habitats conducive to their replication and survival.

## 2. Overview of Cyanophage

Cyanophages play a crucial role in shaping both the local and global ecosystems. Here we extracted the genomes of each fully sequenced cyanophage, deposited in the NCBI database (Table 1). For these 153 cyanophage, in Table 1, we record their taxonomy, host, number of proteins, genes and tRNA and geographic distribution. We explored the annotation of each cyanophage genome and noted 45 regularly occurring AMGs, which may play a key role in altering host metabolism following infection (Table 2). To verify the homology with known genes, we analyzed each AMG in each cyanophage genome using BLAST. Gene functions were deduced through the corresponding literature.

**Figure 1:**
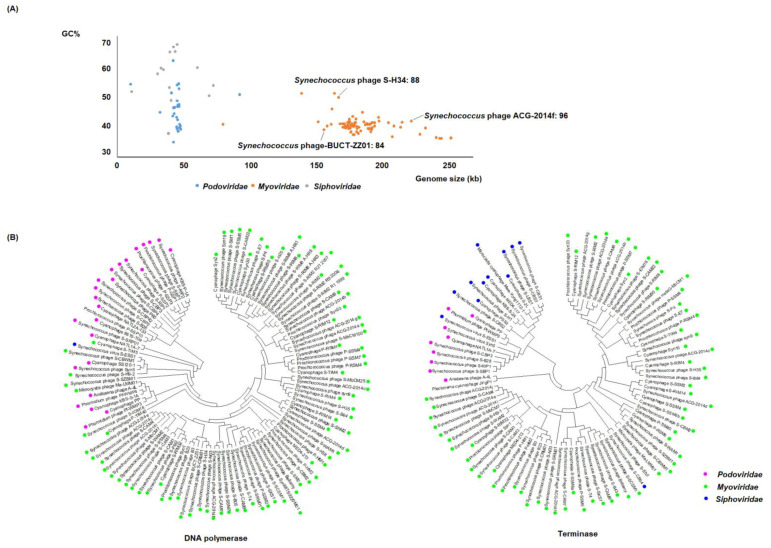
Genomic and phylogenetic features of cyanophage families. (**A**) Scatterplot of the GC (guanine–cytosine) content and genome size. (**B**) Phylogenetic ML tree with related cyanophages based on the nucleotide sequences of DNA polymerase and terminase. The trees were constructed in MEGA version 7 by the ML method.

Cyanobacteriophages can be categorized, according to their morphology, into three families: *Myoviridae* and *Podoviridae* with a GC content of 40% and 46%, respectively, and *Siphoviridae* with the highest GC content (56%). (Figure 1A). Cyanophages belonging to *Myoviridae*, (myocyanophages), have markedly larger genomes (x = 184.18 kb, standard deviation (SD) = 23.04 kb) than those of *Podoviridae* and *Siphoviridae* (x = 44.95 kb, SD = 10.83 kb, x = 42.58 kb, SD = 15.36 kb, respectively), and are speculated to use a different strategy to take over the host.

Recent studies show that the encoded tRNA genes within the myocyanophage can alter the host codon translational efficiency [35,36]. This provides a competitive advantage, enabling the myocyanophage to infect a much broader range of hosts than their podocyanophage and siphocyanophage counterparts [36,37,38]. The divergence of nucleotide sequences from different cyanophages may be seen through comparative genome analysis (Figure 1B). DNA polymerase and terminase are widely distributed in the podocyanophages and myocyanophages. DNA polymerase has not been discovered in most of the siphocyanophages, whereas terminase exists in most of the siphocyanophages. We based the phylogenetic analysis on the nucleotide sequences of the conserved genes, DNA polymerase and terminase, using the maximum likelihood method (ML) (Figure 1B). Generally, close genetic relationship may be seen within families, and a more distinct genetic relationship may be seen between families, though notable exceptions may be observed.

**Figure 2:**
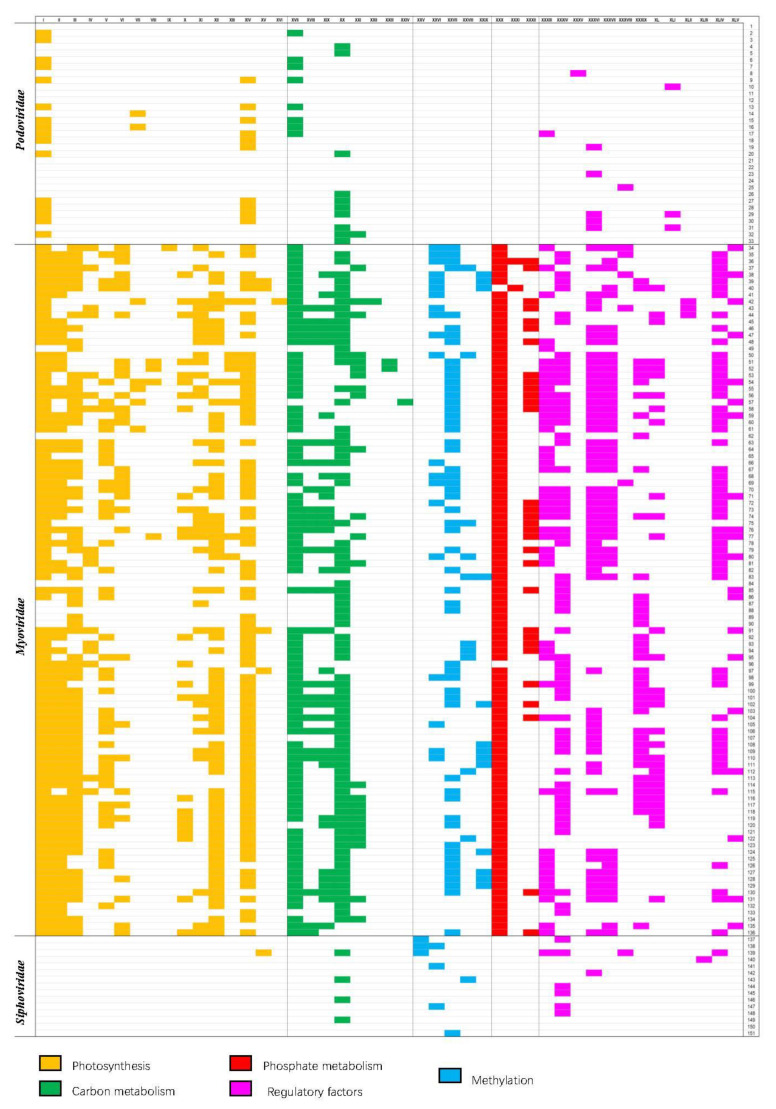
Widespread distribution of AMGs related to photosynthesis and carbon metabolism across the 153 cyanophage AMGs labelled I–XLV and cyanophages labelled 1–151. **AMGs involved in photosynthesis and carbon metabolism**. **I.** Photosystem **II** reaction center protein PsbA/D1, **II.** Photosystem II D2 protein, **III.** 2OG-Fe (II) oxygenase family oxidoreductase, **IV.** Phycocyanobilin: ferredoxin oxidoreductase (PcyA), **V.** Ferredoxin (PetF), **VI.** Phycoerythrobilin lyase (CpeT), **VII.** Phycoerythrobilin synthase (PebS), **VIII.** Phycoerythrobilin, **IX.** NADH dehydrogenase subunit I, **X**. NAD synthetase, **XI.** Plastoquinol terminal oxidase PtoX. **XII.** Plastocyanin (PetE), **XIII.** Heme oxygenase (HO), **XIV.** Hli gene family/high-light inducible protein, **XV.** Thioredoxin, **XVI.** RfaE bifunctional protein, **XVII.** Transaldolase family protein (TalC) in this family, **XVIII.** Glucose 6-phosphate dehydrogenase Zwf, **XIX.** 6-phosphogluconate dehydrogenase Gnd, **XX.** FAD-dependent thymidylate synthase/thymidylate synthase, **XXI.** Carboxylesterase, **XXII.** UDP-glucose 6-dehydrogenase, **XXIII.** Acyl carrier protein ACP, **XXIV.** Fructose-6-phosphate aldolase, **XXV.** Phage host specificity protein, **XXVI.** DNA methylase, **XXVII.** DNA adenine methylase Dam, **XXVIII.** Cytosine methyltransferase, **XXIX.** Type II N-methyl DNA methyltransferase (group alpha), **XXX.** Phosphate-starvation inducible protein (PhoH), **XXXI.** Phosphate-induced stress protein, **XXXII.** Phosphate ABC transporter phosphate-binding protein PstS **XXXIII.** Nrd, **XXXIV.** RNA polymerase sigma factor RpoD/rpoS late transcription sigma factor, **XXXV.** ERF family protein, **XXXVI.** MazG, **XXXVII.** RegA photosynthetic apparatus regulatory protein, **XXXVIII.** Transcriptional regulator, **XXXIX.** late promoter transcriptional accessory protein, **XL.** CP12 carbon metabolic regulator, **XLI.** MarR family protein **XLII.** Translational regulator, **XLIII.** XRE family transcriptional regulator, **XLIV.** CobS/CobO/CobA, **XLV.** Tryptophan halogenase-like gene. **Cyanophage 1.** *Anabaena* phage A-4L, **2.** Cyanophage 9515-10a, **3.** Cyanophage BHS3, **4.** Cyanophage KBS-P-1A, **5.** Cyanophage KBS-S-1A, **6.** Cyanophage NATL1A-7, **7.** Cyanophage NATL2A-133, **8.** Cyanophage PP, **9.** Cyanophage P-SSP2, **10.** Cyanophage SS120-1, **11.** *Phormidium* virus WMP3, **12.** *Phormidium* virus WMP4, **13**. *Prochlorococcus* phage P-GSP1, **14.** *Prochlorococcus* phage P-RSP2, **15.** *Prochlorococcus* phage P-SSP3, **16.** *Prochlorococcus* phage P-SSP6, **17.** *Prochlorococcus* virus PSSP7, **18.** *Synechococcus* phage S-CBP4, **19.** *Synechococcus* phage S-CBP42, **20.** *Synechococcus* phage S-RIP1, **21.** Cyanophage Pf-WMP3, **22.** Cyanophage Pf-WMP4, **23.** *Synechococcus* phage P60, **24.** *Synechococcus* phage MRHenn-2013a, **25**. *Synechococcus* phage S-SRP02, **26**. *Synechococcus* phage S-SRP01, **27**. *Synechococcus* phage S-SBP1, **28**. *Synechococcus* phage S-B28, **29**. *Synechococcus* phage S-CBP1, **30**. *Synechococcus* phage S-CBP2, **31**. *Synechococcus* phage S-CBP3, **32**. *Synechococcus* phage S-RIP2, **33**. *Synechococcus* virus Syn5, **34**. Cyanophage P-TIM40, **35**. Cyanophage S-RIM4, **36**. Cyanophage S-RIM12, **37**. Cyanophage P-RSM3, **38**. Cyanophage S-RIM14, **39**. Cyanophage S-RIM32, **40**. Cyanophage S-RIM50, **41**. Cyanophage S-SSM2, **42**. Cyanophage S-SSM6a, **43**. Cyanophage S-SSM6b, **44**. Cyanophage S-TIM4, **45**. Cyanophage S-TIM5, **46**. Cyanophage Syn2, **47**. Cyanophage Syn10, **48**. Cyanophage Syn30, **49**. *Microcystis* virus Ma-LMM01, **50**. *Prochlorococcus* phage MED4-213, **51**. *Prochlorococcus* phage P-HM1, **52**. *Prochlorococcus* phage P-HM2, **53**. *Prochlorococcus* phage P-RSM4, **54**. *Prochlorococcus* phage P-SSM2, **55**. *Prochlorococcus* phage P-SSM3, **56**. *Prochlorococcus* phage P-SSM4, **57**. *Prochlorococcus* phage P-SSM5, **58**. *Prochlorococcus* phage P-SSM7, **59**. *Prochlorococcus* phage Syn1, **60**. *Prochlorococcus* phage Syn33, **61**. *Synechococcus* phage KBS-M-1A, **62**. *Synechococcus* phage S-B68, **63**. *Synechococcus* phage S-CAM1, **64**. *Synechococcus* phage S-CAM8, **65**. *Synechococcus* phage S-CBM2, **66**. *Synechococcus* phage S-IOM18, **67**. *Synechococcus* phage S-PM2, **68**. *Synechococcus* phage S-RIM2, **69**. *Synechococcus* phage S-RIM8, **70**. *Synechococcus* phage S-RSM4, **71**. *Synechococcus* phage S-ShM2, **72**. *Synechococcus* phage S-SKS1, **73**. *Synechococcus* phage S-SM1, **74**. *Synechococcus* phage S-SM2, **75**. *Synechococcus* phage S-SSM4, **76**. *Synechococcus* phage S-SSM5, **77**. *Synechococcus* phage S-SSM7, **78**. *Synechococcus* phage S-WAM1, **79**. Cyanophage P-RSM1, **80**. Cyanophage P-RSM6, **81**. Cyanophage P-SS1, **82**. *Synechococcus* phage S-WAM2, **83**. *Synechococcus* phage S-CRM01, **84**. *Synechococcus* phage BUCT-ZZ01, **85**. *Synechococcus* phage S-H38, **86**. *Synechococcus* phage S-SRM01, **87**. *Synechococcus* phage S-N03, **88**. *Synechococcus* phage S-H34, **89**. *Synechococcus* phage B3, **90**. *Synechococcus* phage B23, **91**. *Synechococcus* phage S-SCSM1, **92**. *Synechococcus* phage S-H68, **93**. *Synechoccus* phage S-B43, **94**. *Synechococcus* phage S-B05, **95**. *Synechococcus* phage S-SZBM1, **96**. *Synechococcus* phage ACG-2014f, **97**. *Synechococcus* phage S-H9-1, **98**. *Synechococcus* phage S-H9-2, **99**. *Synechococcus* phage SynMITS9220M01, **100**. *Synechococcus* phage S-H25, **101**. *Synechococcus* phage S-P4, **102**. Synechoccus phage S-E7, **103**. *Synechococcus* phage S-B64, **104**. *Synechococcus* phage Bellamy, **105**. *Synechococcus* phage S-H35, **106**. *Synechococcus* phage S-CAM9, **107**. *Synechococcus* phage S-CAM7, **108**. *Synechococcus* phage S-CAM4, **109**. *Synechococcus* phage S-CAM3, **110**. *Synechococcus* phage S-CAM22, **111**. *Synechococcus* cyanophage S-WAM2, **112**. *Synechococcus* cyanophage S-WAM1, **113**. *Synechococcus* phage ACG-2014j, **114**. *Synechococcus* phage ACG-2014i, **115**. *Synechococcus* phage metaG-MbCM1, **116**. *Synechococcus* phage ACG-2014g, **117**. *Synechococcus* phage ACG-2014e, **118**. *Synechococcus* phage ACG-2014d, **119**. *Synechococcus* phage ACG-2014b, **120**. *Synechococcus* phage ACG-2014a, **121**. *Synechococcus* phage S-MbCM100, **122**. *Synechococcus* phage S-MbCM25, **123**. *Synechococcus* phage S-MbCM7, **124**. *Synechococcus* phage S-RIM8 A.HR5, **125**. *Synechococcus* phage S-RIM8 A.HR3, **126**. *Synechococcus* phage S-RIM8 A.HR1, **127**. *Synechococcus* phage S-RIM2 R9_2006, **128**. *Synechococcus* phage S-RIM2 R1_1999, **129**. *Synechococcus* phage S-RIM2 R21_2007, **130**. *Synechococcus* phage Syn30, **131**. *Synechococcus* phage ACG-2014c, **132**. *Synechococcus* phage S-T4, **133**. *Synechococcus* phage S-CBWM1, **134**. *Synechococcus* phage ACG-2014h, **135**. *Synechococcus* phage syn9, **136**. *Synechococcus* phage Syn19, **137**. Cyanophage KBS-S-2A, **138**. Cyanophage MED4-117, **139**. Cyanophage PSS2, **140**. *Microcystis* phage MACPNOA1, **141**. *Prochlorococcus* phage MED4-184, **142**. Cyanophage S-2L, **143**. *Synechococcus* phage S-H1, **144**. *Synechococcus* phage S-LBS1, **145**. *Synechococcus* phage S-CBS1, **146**. *Synechococcus* phage S-CBS4, **147**. *Synechococcus* phage S-CBS3, **148**. *Synechococcus* phage S-CBS2, **149**. *Synechococcus* virus S-ESS1, **150**. *Microcystis* cyanophage vB_MaeS-yong1, **151**. *Microcystis* cyanophage Mwe-Yong1112-1.

The 153 fully sequenced cyanophages from NCBI comprise 33 podocyanophages, 103 myocyanophages, 15 siphocyanophages, and 2 unclassified phages. We identified and discounted all genes associated with phage replication from these sequences. In the remaining sequence data, a palette of 45 AMGs that may play a key role in altering host metabolism following infection could be seen to occur frequently. Though a few AMGs were seen to occur in only one virus, many were seen to be widely distributed across the majority of viruses that were analyzed (Figure 2), implying the importance and evolutionary advantage of the roles that they play. The 45 AMGs encoded within the cyanophages were all homologs of genes found within cyanobacteria that play a key role in cyanobacterial metabolism. In addition to genes encoding the assembly of new cyanophage genes relating to enhancing photosynthesis that dominate, genes relating to central carbon metabolism, phosphate metabolism, methylation, and regulation are prevalent. Genes with sequence similarity to known halogenases are also abundant. This global analysis and distribution of the AMGs are presented in Figure 2. The AMGs and their potential role as the cyanophage infects the host cyanobacterium are reviewed in the following sections.

After infection, cyanophage AMGs are expressed, impacting photosynthesis, carbon metabolism, phosphate metabolism, and regulation. The resulting energy and flux of precursors are redirected toward phage DNA synthesis and capsid production. The counterparts of numerous AMGs linked to photosynthesis and central carbon metabolism may be seen in the genomes of myocyanophages. This is undoubtedly their origin. As shown above (Figure 1A), myocyanophages generally have considerably larger genomes than the other cyanophages and possess a long contractile tail enabling the delivery of this much larger genome into bacteria. This tail is sheathed and operates via a contractile mechanism, resulting in the highly efficient injection of its DNA into the host. As a result of housing a much larger genome, the heads of myocyanophages are larger than those of other cyanophages. This larger casing, more sophisticated injection machinery, in combination with the much larger genome, places a greater metabolic burden on the host. Correspondingly, almost all of the myocyanophages can be seen to carry multiple AMGs that potentially force the upregulation of photosynthesis to sequester further carbon and the upregulation of carbon metabolism to generate the substantial additional energy needed. Curiously, two exceptions emerge: *Synechococcus* phage-S-H34, (Figure 2, entry 88), an outlier in terms of its much higher GC content, appears not to have any AMGs related to photosynthesis. *Synechococcus* phage-BUCT-ZZ01 (Figure 2, entry 84), one of the smaller myocyanophage, appears to not have any AMGs related to glycolysis. The availability of phosphate is essential to the generation of new DNA as the phage replicates. So far, the myocyanophage alone may be seen to possess AMGs linked to the modulation of phosphate metabolism. It is puzzling that one of the larger myocyanophages, *Synechococcus* phage-ACG-2014f, does not have any AMGs related to phosphate metabolism (Figure 2, entry 96).

In analyzing the data, we sought first to explore genes linked to photosynthesis, with the question in mind as to what extent might viral infection enhance photosynthesis and thus carbon capture and oxygen production?

Of the 153 viral DNA sequences analyzed, almost all could be seen to contain AMGs encoding proteins with a role in photosynthesis, with the potential to augment or control the infected host’s photosynthetic activity. Only seven of the candidates did not clearly show the presence of such a gene. AMGs related directly to the enhancement of photosynthesis, including genes encoding the production of the photosystem II reaction center protein, the production of the high-light inducible protein, the antennae protein as well as the production of photosynthetic pigments, were clearly visible in many of the viruses.

## 3. The Photosynthetic Machinery Used by Cyanobacteria

**Figure 3:**
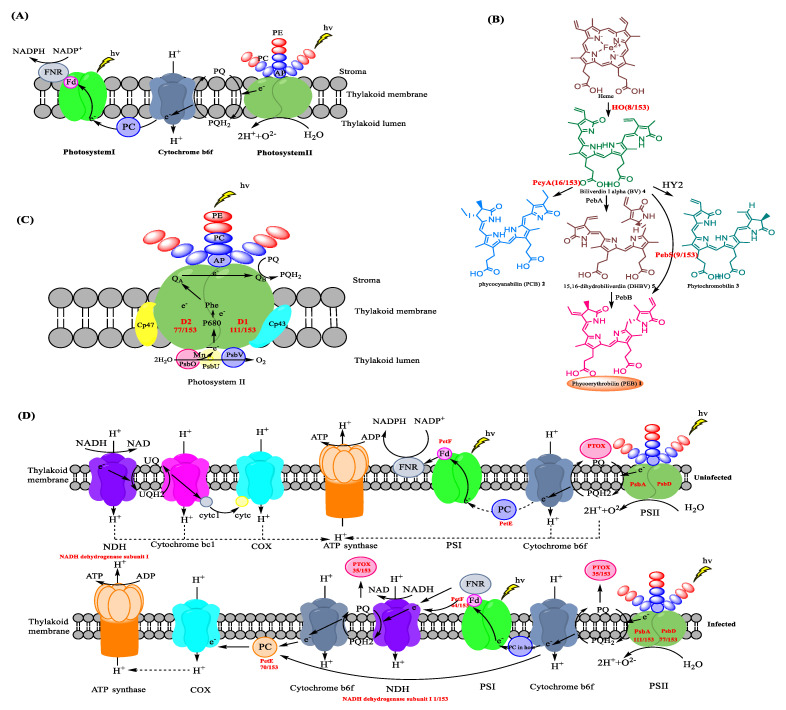
Photosynthesis affected by phage infection. (**A**) The three multicomponent photosynthetic complexes used by cyanobacteria [43]. (**B**) Biosynthesis of the chromophores Phycoerythrobilin (PEB) [44], Phycocyanobilin 2, and Phytochromobilin 3. Enzymes that we note to be encoded by AMGs, through our analyses of cyanophage genomes, are depicted in an orange sphere. For the full list of cyanophages that encode HO, PcyA, PebS, and PEB, see Figure 2 entries XIII, IV, VII, and VIII, respectively. PEB could also be found in four myocyanophage genomes. (**C**) The polypeptide composition of and electron transport in the photosystem II complex [45]. AP: Allophycocyanin, PE: phycoerythrin, PC: phycocyanin, P680: reaction center chlorophyll a dimer, Phe: pheophytin, Q_A_: plastoquinone tightly bound to the D2 protein, Q_B_: exchangeable plastoquinone bound to the D1 protein, PQ: plastoquinone and PQH_2_: plastohydroquinone. (**D**) Modifications to photosynthetic pathways caused by phage infection [46]. The photosynthetic machinery and mechanisms of uninfected (**top**) and phage-infected cyanobacteria (**bottom**). Cyanophage genes involved in the photosystem are gene *psbA* encoding photosystem II reaction center protein D1, *psbD* encoding photosystem II D2 protein, *ptoX* encoding plastoquinol terminal oxidase (PTOX), *petF* encoding ferredoxin, and *petE* encoding plastocyanin and the NADH dehydrogenase gene (shown in red bold font). NDH, NAD(P)H dehydrogenase. COX, cytochrome c oxidase. PTOX, plastoquinol terminal oxidase. PC, plastocyanin.

Cyanobacteria have evolved to adapt quickly and are able to efficiently capture light across a wide range of different wavelengths using a range of photosynthetic pigments including carotenoids, chlorophylls, and the tetrapyrrolic derivatives of chlorophylls. Cyanobacteria utilize photosynthetic machinery comprising three multi-subunit complexes, photosystems I and II (PSI, PSII) [39] plus the additional phycobilisomes (PBS) that attach to the cytoplasmic surface of thylakoid membranes and closely interact with PSII [40] (Figure 3A). The PBS comprise phycolipoproteins (PBPs) together with linker proteins. The PBPs are water soluble and brightly colored. The color arises from the attachment of a series of tetrapyrrole chromophores via a thioester linkage to a cysteine residue within the PBP [41]. The PBPs are subdivided into three groups relating to the energy they absorb and to the tetrapyrrole chromophore. The allophycocyanins (APCs) are chromophoric proteins that absorb low-energy light and make up much of the core of the PBS. At the base of the rods, next to the core, are the phycocyanins (PCs), which absorb intermediate-energy light, and at the far end of the rod are the phycoerythrins (PEs), which absorb high-energy light [42].

Marine and freshwater cyanobacteria, red algae, and cryptophytes all produce and use a subclass of the light-harvesting pigment phycoerythrin (PE) that is pink. This open-chain tetrapyrrole pigment is called phycoerythrobilin (PEB) **1.** In addition to PE, marine cyanobacteria also use the pigment phycourobilin (PUB), which is an isomer of PEB. The PE produced by freshwater cyanobacteria contains solely PEB, in contrast to their marine counterparts, which carry both PUB and PEB. Using PEs, within the PBS, cyanobacteria can absorb light between 450 and 700 nm at depths deeper than 200 m in the ocean where low light levels render chlorophyll ineffective.

### 3.1. AMGs with the Potential to Impact the Biogenesis of the PBS

Photosynthetic pigments are biosynthesized and then formed into the PBS, an assemblage of chromophorylated proteins anchored to thylakoid membranes. In cyanobacteria, the biosynthesis of PEB **1**, as well as the associated pigments phycocyanabilin **2** and phytochromobilin **3**, starts with the heme oxygenase (HO)-mediated cleavage of heme to an open-chain product biliverdin IXa (BV) **4** through an aerobic reaction [47,48]. Of the 153 sequenced cyanophage genomes, 8 could be seen to contain a gene encoding an additional copy of HO (XIII in Figure 2 and Figure 3B. Table 2).

Ferredoxin oxidoreductase, encoded by *pebA*, is employed to catalyze the two-electron reduction of biliverdin IXa (BV) **4** to 15,16 dihydrobiliverdin (DHBV) **5** [49]. The conversion of DHBV **5** to phycoerythrobilin (PEB) **1** is subsequently mediated by PebB (Phycoerythrobilin: ferredoxin oxidoreductase). The direct conversion of BV **4** to PEB **1** mediated by PebS (phycoerythrobilin synthase), is also possible. Intriguingly, of the 153 sequenced cyanophage analyzed, 9 of them may be seen to contain a gene encoding PebS (VII in Figure 2 and Figure 3B; Table 2).

Phycocyanobilin:ferredoxin oxidoreductase (PcyA) belongs to the ferredoxin-dependent bilin reductase (FDBR) family [50,51] and mediates the conversion of BV **4** to phycocyanabilin (PCB) **2**. Through the catalysis of PcyA, the substrate BV **4** can be converted to phycocyanobilin (PCB) directly [50,52]. The gene encoding PcyA may be seen in 16 of the 153 sequenced genomes (IV in Figure 2 and Figure 3B, Table 2). Once the chromophores are biosynthesized, they are integrated into the PBPs through the formation of thioester links with cysteine residues. They are then assembled into the PBS. A putative phycobiliprotein lyase (CpeT) [8], likely involved in supporting the attachment of PEB to the apo-phycobiliprotein, provides a scaffold for the pigment to ensure binding in the right conformation to facilitate correct stereospecific ligation to a conserved cysteine residue within the phycobiliprotein. CpeT belongs to the so-called T-type lyases, and phycocyanobilin-specific homologs of CpeT (i.e., CpcT) were shown to specifically serve the position equivalent to cysteine 153 in the -subunit of phycocyanin (PC) [53]. Therefore, the analogous CpeT lyases may serve the Cys-153 of PE. It has been speculated that their expression also contributes to phage fitness by enhancing the light-harvesting capacity. In this regard, the cyanophage S-PM2 was shown to induce increased synthesis of the light-harvesting PE in *Synechococcus* sp. WH7803 during infection [54]. CpeT was found in 31 of the 153 sequenced genomes (VI in Figure 2 and Figure 3B, Table 2)

Notably, it is only the myocyanophages that are seen to carry the AMGs HO, PcyA, and PebS responsible for the biosynthesis of photosynthetic pigments. Similarly, it is only the myocyanophages that carry the gene CpeT, responsible for the ligation of the photosynthetic pigments to the light-harvesting protein complex. The only exception seen in the set of viruses analyzed is that PebS is also encoded by the single podovirus cyanophage 9515-10a. Perhaps it is that among the three families only the myocyanophages have adapted to benefit from enhancing their host cell’s biosynthetic capability, or the other two families of much smaller phages (podocyanophages and siphocyanophages) do not require increased carbon flux.

### 3.2. AMGs with the Potential to Repair Photosystem II following Oxidative Damage

As a large common protein complex, photosystem II (PSII) extracts the electrons from water and releases molecular oxygen [55]. Polypeptide D1 and D2, encoded by *psbA* and *psbD*, respectively, form the core heterodimer of the PSII reaction center. Together with the necessary cofactors, PSII enables the generation of a strong oxidant that can be used to extract electrons from water (Figure 3C). The generation of this strong oxidant results in abundant levels of molecular oxygen, which can cause photo-oxidative damage. The polypeptides D1 and D2, especially D1, are particularly vulnerable to photodamage [56]. This could irreversibly inhibit the function of PSII. Stress conditions, such as high light, high salt concentration, or high or low temperatures, may exacerbate the damage [57]. To counteract the damage, cyanobacteria have evolved a PSII repair mechanism, whereby damaged D1 can be degraded and complemented by the newly synthesized D1 polypeptides. If the synthesis of D1 polypeptides can complement the damage, then photosynthesis would not be affected. However, if the synthesis of D1 polypeptides cannot complement the damage, photoinhibition occurs, and the growth of cells would be affected. In a recent study, the phototrophs treated with protein synthesis inhibitors were shown to inhibit the synthesis of D1 polypeptides and result in photoinhibition [58,59]. This demonstrates that D1 peptide synthesis is especially important for the PSII repair cycle against photoinhibition. Different species and growth conditions can affect the half-life of D1 polypeptides. For example, in higher plants, the half-life of D1 is about 2–6 h. The half-life of D1 in *Synechococcus* WH7803 is about 3–4 h [57].

Photodamage to the D1 polypeptide is unavoidable, regardless of the prevailing light conditions. Most of the investigated viruses contain AMGs encoding D1 (111/153). Based on our analyses, 16/33 podocyanophages and 95/103 myocyanophages contain a copy of *psbA.* AMGs encoding D2 were also prevalent in the myocyanophages analyzed (78/103) but absent in podoviridae and siphoviridae.

Previous studies have indicated that shortly after infection, archetypal myovirus T4 can block host DNA replication, transcription, and translation and degrade the host DNA [57]. In *Synechococcus* WH7803 cells, S-PM2 infection exerts a similar effect on the host. One effect of infection would be to prevent the D1 protein re-synthesis. Indeed, the transcription of the gene *psbA* is markedly and progressively reduced following S-PM2 infection [57]. Thus, compared with uninfected cells, the infected cells would be more sensitive to photoinhibition. This also explains why the acquisition of a copy of the *psbA* gene would confer a fitness advantage to the phage. By encoding its own copy of D1, S-PM2 maintains the PSII repair cycle after the synthesis of D1 proteins in host cells have been shut down, thus permitting the continuation of photosynthesis and providing the necessary energy for phage replication.

It could be postulated that after infection, more *psbA* genes need to be transcribed to complement the loss of D1 polypeptides. The *psbD* AMG-encoding D2 polypeptide was found in 78 of the 153 cyanophage that we analyzed.

In *Prochlorococcus* myoviruses P-SSM2 and P-SSM4 [60], there are putative promoter and transcriptional terminators flanking the genes *psbA* and *psbD*, indicating they can be expressed autonomously [61]. Gene *psbA* and the essential and highly expressed phage capsid genes in the podocyanophage P-SSP7 have the same promoters and terminators, enabling simultaneous transcription, implying that *psbA* has become an integral part of phage genomes [61].

### 3.3. AMGs with the Potential to Impact Electron Transport

Plastocyanin (PC), encoded by the gene *petE*, can transfer the electron flow from cytochrome b6*f* to photosystem I in cyanobacteria (XII in Figure 2 and Figure 3D). However, studies indicate that PC encoded by cyanophages may have a different function [50]. The hydrophobic core region of PC directly follows the signal peptide of the cyanophage PC without a C-terminal domain [62]. The N-terminal signal peptide of the cyanophage PC is also modified. Compared with the host, cyanophage PC also contains many unique amino acid residues [38]. In cyanobacteria, the isoelectric point (pI) is essential for the interactions of PC and PSI. However, the cyanophage PC has a substantially different pI compared to the host (Krukal–Wallis: H1 = 50.21, *p* < 0.001). All of these findings may imply a potential functional difference in PC between the host and cyanophage. After the acceptance of electrons from the cytochrome b6*f* complex, cyanophage PC may not reduce the PSI, but could, instead, transfer electrons to cytochrome c oxidase (COX) directly [63]. Following the reduction of COX, the proton is transferred across the thylakoid [59]. Through this electron transfer pathway, electrons could accumulate for ATP synthesis by ATP synthase. Furthermore, the over-production of the PQ pool (plastoquinol, reduced; plastoquinone, oxidized) caused by the cyanophage infection may be alleviated in this way, which could prevent the photosystem from undergoing photoinhibition [38]. Of the 153 sequenced cyanophage genomes, 70 of them contain the PC-encoded gene *petE* and are all from myocyanophages. According to a recent study, following phage infection, the COX expression level may be enhanced, which may support the above hypothesis for the role of this complex in accepting electrons from the plastocyanin (PC) pool during infection [46].

Cyanophages can also encode type I NAD(P)H dehydrogenase, also known as the NDH -1 complex, which can transfer electrons to quinol and participate in photosynthesis and CO_2_ uptake [64]. Through metagenomic analysis, cyanophages have been shown to have the *ndhI* gene-encoded NADH dehydrogenase subunit 1 [65], the *ndhD*-gene encoded NADH dehydrogenase subunit 4 [66], as well as the *ndhP* gene-encoded single transmembrane small subunit of the NDH-1 complex [67]. In the genome of P-TIM40, we also identified the *ndhI* gene (IX in Figure 2 and Table 2) that encoded a Fe-S protein. The cysteine residues that directly bind the two Fe–S centers (N6a and N6b) of the protein are completely conserved in the suspected cyanophage homolog, suggesting that NdhI may play a key role in electron transfer through the complex directly to reduce plastoquinol [65]. Electrons are then transferred from plastoquinol to PC through the cytochrome b6*f* complex. To date, the mechanism of electron donation to NdhI has not been clearly understood, although there are several hypotheses [64,68,69]. It is also difficult to know whether there are any functional differences between cyanobacterial NdhI and the cyanophage NdhI.

In cyanobacteria, ferredoxin (Fd), encoded by the *petF* gene, can transfer electrons from photosystem I to ferredoxin NADP^+^ reductase (FNR), which, in turn, reduces NADP to NADPH for carbon fixation (Figure 3D). However, according to some hypotheses, electron flow may be redirected from Fd to the NDH-1 complex after infection [38]. Furthermore, the ferredoxin-dependent bilin reductases (FDBRs) can catalyze BV reduction, where Fd provides electrons for this reaction [70]. As a class of radical enzymes, FDBRs are able to catalyze the biosynthesis of both PEB and PCB [50,70,71]. Among the 153 sequenced cyanophage genomes, the gene *petF* encoding Fd was found in 44 of them. In a recent study, the transcriptome difference between a cyanophage (P-HM2) and its host (*Prochlorococcus* MED4) throughout infection was analyzed. Following infection, the host transcription levels of photosystem I assembly protein Ycf37, FNR, and other translation-related-genes were diminished, suggesting that these could affect the reducing power flow during the Calvin cycle carbon fixation [46].

A putative plastoquinol terminal oxidase (PTOX) is commonly found in cyanophage genomes [21]. Of the 153 sequenced cyanophages, PTOX is observed in 35 of these genomes, notably only pertaining to a subset of cyanophages known as myocyanophages. PTOX is thought to be a safety valve in these phages to protect photo electron transfer (PET) proteins from photodamage, especially under stressful conditions [38]. When the PQ pool is over reduced, often, as a result of high light or low iron conditions, electrons can be donated to oxygen through PTOX [72,73]. This is a particularly important flow of electrons when considering the viral-induced inhibition of PSI and FNR [46]. This effect could explain why PTOX expression levels are seen to be upregulated in cyanophage-infected cells and exist in many cyanophages.

High-light-inducible proteins (HLIPs) can also be found in many cyanophage genomes (90/153). In cyanophages, the function of HLIPs has been an enigma until recently, as very little information has been available on the role of HLIPs in the infection process. However, a recent knockout study of *hli* genes in *Synechocystis* sp. PCC6803 showed that knockout mutants were sensitive to high irradiances [74,75]. HLIPs, which contain one membrane-spanning helix and are located in the thylakoid [74], could associate with both PSII [76] and PSI [77]. The study demonstrated that HLIPs were constructive to PSI stabilization [77], tetrapyrrole biosynthesis regulation [78], the transient store of chlorophyll during PSII repair and assembly [79], and excess excitation energy dissipation [38,75]. It is likely that cyanophage HLIPs have similar functions to cyanobacterial HLIPs.

There are many genes involved in photosynthesis that are encoded within cyanophage genomes (Table 1). In addition to this, the expression levels of some host-encoded photosynthesis genes (such as COX) increased after infection. Transcription levels of ferredoxin NADP^+^ reductase (FNR) and some photosystem I proteins were diminished, leading to the reduction of NADPH biosynthesis. More electrons were transferred to COX through the cyanophage PC, meaning more protons could accumulate for ATP synthesis, resulting in more energy for cyanophage DNA biosynthesis.

## 4. AMGs with the Potential to Impact Central Carbon Metabolism

**Scheme 2:**
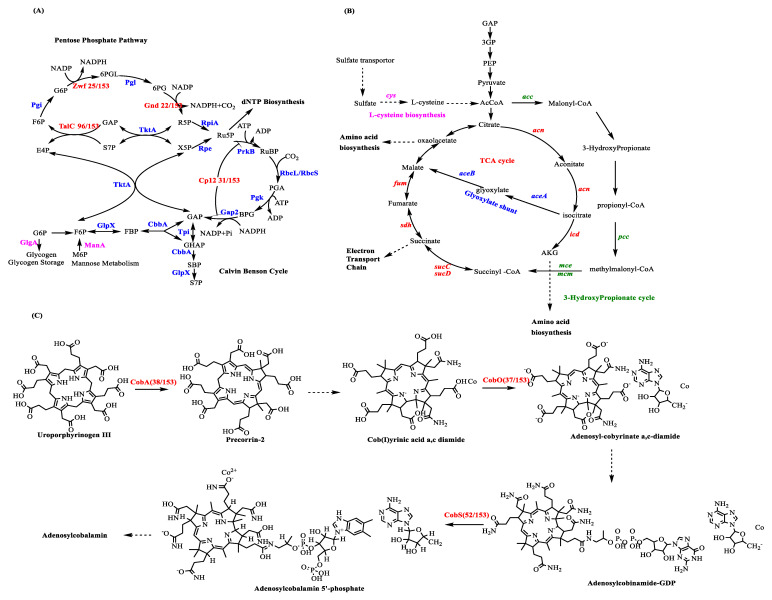
AMGs involved in central carbon metabolism. (**A**) Cyanophage genes involved in the Calvin–Benson cycle and pentose phosphate pathway. After infection, the expression of cyanophage gene *cp12* can inhibit the Calvin cycle and carbon fixation. The expression of cyanophage genes *talC*, *zwf*, and *gnd* can enhance the PPP. Pi, phosphate; CbbA, fructose-1,6-bis P/sedoheptulose-1,7-bis-P aldolase; GlpX, fructose-1,6/sedoheptulose-1,7-bisphosphatase; Pgi, P-glucose isomerase; Pgk, P-glycerate kinase; Pgl, 6-P-gluconolactonase; PrkB, P-ribulokinase; RbcLS, ribulose-1,5-bis-P carboxylase/oxygenase; Rpe, ribulose-5-P epimerase; RpiA, ribulose-5-P isomerase; TktA, transketolase; Tpi, triose-P isomerase; BPG, 2,3-bis-P-glycerate; DHAP, DHA P; E4P, erythrose 4-P; FBP, fructose 1,6-bisP; F6P, fructose 6-P; GAP, glyceraldehyde 3-P; G6P, glucose 6-P; PGA, 3-P-glyceric acid; R5P, ribose 5-P; RuBP, ribulose 1,5-bis-P; Ru5P, ribulose 5-P; SBP, sedoheptulose 1,7-bis-P; 6PG, 6-P-gluconate; 6PGL, 6-P-gluconolactone; S7P, sedoheptulose 7-P; and X5P, xylulose 5-P. (**B**) Cyanophage genes involved in L-cysteine biosynthesis (Purple), Tricarboxylic acid (TCA) cycle (Red), glyoxylate shunt (Blue), and 3-hydroxypropionate (Green). The genes (~3-fold) of the TCA cycle were those involved in the glyoxylate shunt. The glyoxylate shunt was used to increase the cellular reducing power and ATP for phage synthesis. (**C**) Cyanophage genes involved in the adenosylcobalamin (cofactor B12) biosynthesis pathway.

In cyanobacteria, energy (ATP) and reducing power (NADPH) produced by ATP synthase and ferredoxin NADP^+^ reductase (FNR) during photosynthesis are mostly used for carbon fixation in the Calvin cycle [80]. However, cyanophage infection can drastically alter the energy flow. This research reveals that more than 20% of cyanophage genomes contain a *cp12* gene, which encodes for a Calvin cycle inhibitor. In cyanobacteria, the Calvin cycle is a key pathway for carbon fixation. Two essential enzymes of the Calvin cycle are phosphoribulokinase (PRK) and glyceraldehyde-3-phosphate dehydrogenase (GADPH/Gap2). The first step of the cycle, PRK, catalyzes the conversion of D-ribulose 5-phosphate (Ru5P) to D-ribulose 1,5-biphosphate (RuBP), consuming ATP [81]. GADPH catalyzes the conversion of D-glycerate 1,3-biphosphate (BPG) to the end product glyceraldehyde 3-phosphate (GAP), consuming NADPH (Scheme 2A). The Calvin cycle inhibitor CP12 can inhibit the function of these two enzymes by redirecting the carbon flux from GAP synthesis to the pentose phosphate pathway (PPP) and can reduce the consumption of ATP and NADPH. In a study of CP12, particularly in the light and dark cycle of *Prochlorococcus*, researchers found that the *cp12* gene was maximally expressed at night, and in that in dark cycle, carbon flux was directed from the Calvin cycle to PPP in *Prochlorococcus* [82].

In addition to the Calvin cycle, PPP also plays a key role in central carbon metabolism in cyanobacteria [63]. Three PPP enzymes were found in the sequenced cyanophage genomes, i.e., transaldolase (*talc*, 96/153), glucose-6-phosphate dehydrogenase (*zwf*, 25/153), and 6-phosphogluconate dehydrogenase (*gnd*, 22/153) (Scheme 2A). The sequence of TalC is substantially different from that of its host homolog TalB. Through multiple sequence alignment analysis, when compared with TalB, TalC contained numerous deletions; however, there were no changes in the active site residues that were important for catalysis [83,84]. Through activity analysis, the product of the genes *talC* and *talB* could catalyze the conversion of sedoheptulose 7-phosphate (S7P) and GAP to erythrose 4-phosphate (E4P) and fructose 6-phosphate (F6P) (Scheme 2A) [63]. Based on a comparison of the kinetic properties of TalCs with TalBs, the phage transaldolase may have more advantages than their host homologs to enhance PPP in the host during the infection [63].

The PPP gene *zwf*, encoding glucose-6-phosphate dehydrogenase (G6PDH), catalyzes the conversion of glucose-6-phosphate to ribulose 5-phosphate and produces NADPH and CO_2_. The 6-phosphogluconate dehydrogenase, encoded by *gnd*, catalyzes the conversion of gluconate 6-phosphate to gluconolactone 6-phosphate and produces NADPH. Currently, there are only two known enzymes shown to produce NADPH in the PPP. However, until now, little research has been done to explore the cyanophage *zwf* and *gnd* genes. This study shows that *zwf* and *gnd* are present in 25/153 and 22/153, respectively, of the sequenced cyanophage genomes.

The expression of the phage *talC*, *gnd*, and *zwf* genes may augment the PPP and produce more NADPH and the R5P that phages need for dNTP biosynthesis [63]. It is likely that after infection more NADPH would be directed toward phage DNA synthesis through the expression of these four phage genes. In the same study, researchers also found that after infection, the host NADPH/NADP ratio increased twofold, which was consistent with the inhibition of the Calvin cycle and the enhancement of PPP [63]. In a recent study [85], about 4 h infection with phage S-PM2 resulted in the cessation of CO_2_ fixation in *Synechococcus* sp. WH7803. For phage S-RSM4, after about 2 h infection, the CO_2_ fixation in WH7803 was stopped. This is consistent with our analysis. In addition, with the phage genes *cp12 gnd*, *zwf*, and *talc*, S-RSM4 can suspend the CO_2_ fixation of the host faster than phage S-PM2 without these genes.

Through metagenomics analysis, the glycogen biosynthetic gene encoded by the *glgA* gene was identified from the viromes isolated from the Pacific Ocean [86]. The *glgA* gene-encoded enzyme catalyzes the conversion of G6P to glycogen, suggesting that virus infection may induce a starvation response in the host and direct more carbon flux to the glycolysis pathway [87]. The mannose-6-phosphate isomerase, encoded by *manA* and involved in mannose metabolism, was also identified [86]. Mannose-6-phosphate isomerase is known to convert M6P to F6P for use in glycolysis [88] (Scheme 2A).

Global warming has considerable implications for aquatic ecosystems, triggering various changes. With rising water temperature, dissolved oxygen levels can drop, and changes in both pH and salinity can occur [89]. These changes directly impact cyanobacteria, with rising temperatures and CO_2_ levels likely to promote cyanobacterial blooms [90]. There is potential for these blooms to, once again, bring balance, restoring depleted oxygen levels, and to an extent, provide a screen, shading the underlying water from direct sunlight. However, these blooms are limited by nutrient availability. Increasing cyanobacterial levels are likely to result in increased populations of phages. Lysis caused by phage infection results in the re-release of limiting nutrients, enabling the cycle to continue and possibly enabling the further restoration of restored oxygen.

In a recent study, the transcription levels of SLC13 permease, sulfate adenylyl-transferase, adenylyl-sulfate kinase, phosphoadenosine phosphosulfate reductase, assimilatory sulfite reductase, and cysteine synthase A *O*-acetylserine sulfhydrylase were increased after infection of *Pseudoalteromonas* by cyanophage HP1. These genes are involved in the assimilatory sulfate reduction pathway and the L-cysteine biosynthesis pathway (Scheme 2B) [91,92]. Researchers posited that the synthesis of cysteine in infected cells provided more energy because cysteine can be degraded to acetyl-CoA for energy production in the TCA cycle [93]. From the Pacific Ocean Virome dataset, components of the TCA cycle have been identified including aconitase (*acn*), isocitrate dehydrogenase (*icd*), 2-oxoglutarate dehydrogenase (*sucABCD*), isocitrate lyase, succinate dehydrogenase (*sdh*), and fumarate hydratase (*fum*). Malate synthase A (*aceAB*), involved in the glyoxylate shunt, was also identified [86]. In the host *Pseudoalteromonas* infected by cyanophage HP1, the most highly expressed genes (~3-fold) were *aceA* and *aceB* involved in the glyoxylate shunt [91]. The glyoxylate shunt, which is used to increase cellular ATP synthesis and reducing power, is commonly observed under various stressors [94,95].

In addition, two viral gene families were identified from the Pacific Ocean Virome dataset. The fatty acid oxidation complex (*fadB*), the long-chain fatty acid transporter (*fadL*) involved in the fatty acid metabolic pathway, acetyl-CoA carboxylase (*acc*), propionyl-CoA carboxylase (*pcc*), and methylmalonyl-CoA epimerase/mutase (*mcm*) are involved in the 3-hydroxypropionate (3HP) cycle. Considerably more energy can be generated by fatty acid oxidation, and TCA cycle intermediates can be balanced during the infection process. Carbon flux can also be redirected from fixation to energy production by the genes *acc*, *pcc*, *mce*, and *mcm* involved in the 3HP cycle. Through this reprogramming, the carbon and nitrogen cycles may also be influenced (Scheme 2B) [96].

All these changes in central carbon metabolism are consistent with the hypothesis that viruses can redirect carbon flux away from carbon fixation pathways towards dNTP and energy production, which can be used for the generation and assembly of cyanophages.

## 5. AMGs with the Potential to Impact Regulatory Factors

Among the 153 sequenced cyanophage genomes, 65 cyanophages possess putative sigma factors (RpoD/RpoS) (Figure 2). The cyanophage putative sigma factors have high amino acid sequence homology with σ70-type sigma factors in cyanobacteria [97]. As a transcription initiator, the sigma factor binds to the specific sequence and regulates gene transcription. The metabolism, growth, and phenotype of bacteria are affected by the expression of sigma factors. In *Escherichia coli*, there are several sigma factors, including σ70 (RpoD), which is responsible for exponential growth, σ38 (RpoS), which is a stationary phase sigma factor, and the flagellar sigma factor σ28 (RpsD) [98,99]. Cyanobacteria also possess sigma factors in the σ70 family, of which there are three groups categorized upon their amino acid sequences, including sigma factors that control the expression of essential cell growth genes [100], sigma factors that are not essential for cell survival, and other σ70 factors. Furthermore, there are several other putative sigma factors such as σ38 in cyanophage genome sequences. In a recent study, the carbon metabolism in *Synechococcus elongatus* PCC 7942 was modified through the expression of putative sigma factors from *Synechococcus* phages. After the expression of putative RpoD from phage S-CBS2 and putative RpsD4 from phage S-CBS3, the growth of *S. elongatus* PCC 7942 was enhanced noticeably. Through metabolome analysis, the acetyl-CoA concentration was found to increase 3.2-fold and 1.9-fold following expression of the putative RpoD from S-CBS2 and the putative RpsD4 from S-CBS3, respectively [97]. It is possible that the host carbon metabolism can be affected by sigma factors encoded by the cyanophages after infection.

Some genes involved in cofactor B12 synthesis have also been discovered in the sequenced cyanophage genomes including the genes *cobA* (38/153), *cobO* (37/153), and *cobS* (52/153). As an ancient enzyme, ribonucleotide reductase (RNR) reduces ribonucleotides to deoxyribonucleotides before DNA synthesis. Depending on their interaction mechanism with oxygen and the different ways for the generation of thiol radicals, RNRs can be grouped into three classes through their reactivity with O_2_ [101]. Class I RNRs are O_2_-dependent; class II RNRs are O_2_-independent; and class III RNRs are O_2_-sensitive. Adenosylcobalamin (cofactor B12) is a key cofactor of class II RNRs that are commonly observed in cyanophages and cyanobacteria. Genes *cobA*, *cobO*, and *cobS*, which were found in the sequenced cyanophage genomes, also play a key role in the adenosylcobalamin (cofactor B12) biosynthesis pathway (Scheme 2C) [102].

The gene *mazG* encoding dNTP pyrophosphatase activity, MazG, was found in 64 of the 153 sequenced cyanophage genomes (Figure 2). In *E. coli*, MazG acts on the signaling nucleotide guanosine tetraphosphate (ppGpp) with one-third of the *E. coli* genome being regulated by MazG [103]. As a regulator, MazG responds to nutrient stress and programmed cell death [104,105]. In cyanophages, as a global transcriptional regulator through the modulation of ppGpp levels, MazG regulates many metabolic pathways and may also extend the cell survival period under phage infection stress [106]. However, MazG has high non-canonical NTP specificity, indicating the need for the identification of the substrate for each MazG by solving crystal structures and activity analysis [107]. The exact function and substrate of cyanophage MazG would, therefore, need to be cautiously interpreted [21].

As a translational repressor, the RegA protein regulates the expression of several phage early mRNAs in the bacteriophage T4 [108]. In the sequenced cyanophage genomes, 48/153 RegA proteins were observed. In the process of bacteriophage T4 infection, DNA polymerase accessory proteins and membrane proteins of undefined functions are regulated by RegA. These proteins, affected by gene *regA*, may all function in T4 DNA metabolism [109]. Also, the RegA protein may inhibit translation by binding to target mRNAs and blocking the formation of the ribosome initiation complex [110].

The ribonucleoside diphosphate reductase reduces ribonucleosides to ribonucleotides, which is the rate-limiting step of DNA synthesis [111]. *Nrd* genes are found across a wide range of coliphage [112] and environmental [113] phage genomes, with 48 of the 153 sequenced cyanophage genomes containing *nrd* genes. In a recent study where the marine host *Pseudoalteromonas* bacterium was infected by two different viruses, siphovirus PSA-HS2 and podovirus PSA-HP1, the temporal fold value of gene nrdA changed from 4 to 205 and frm 2 to 239, respectively, despite the fact that the *nrd* genes were not found in these two phages [68]. This suggests that ribonucleoside diphosphate reductase plays a key role in phage DNA synthesis after infection.

Twenty of the sequences contained genes that potentially encode flavin-dependent halogenases (FDHs). This group of enzymes is known to introduce a halogen into molecules in the form of a carbon–halogen bond. Halogenated molecules exhibit a wide range of biological activities, bioavailability, and metabolic stability [114,115]. This bond has been shown to increase the thermal, oxidative stability, and permeability of a compound compared with the non-halogenated molecule [116]; thus, a host’s metabolite activity and stability could be enhanced by halogenation resulting from cyanophage infection.

## 6. AMGs with the Potential to Impact Phosphate Metabolism

As a scarce resource in the oceans, phosphorus is often thought to be a growth-limiting factor for cyanobacteria [117]. Phosphorus also plays a key role in phage replication. Therefore, it is not surprising that 101 phosphate-inducible *phoH* genes and 31 phosphate-uptake *pstS* genes were identified in the sequenced cyanophage genomes (Figure 2). The gene *phoH* is widely distributed in cyanobacteria, eubacteria, and archaea [118]. This gene is induced under phosphate stress in *E. coli* [119] although its function has not yet been experimentally verified. Bioinformatic analyses have shown that *phoH* genes are likely part of a multi-gene family with different functions, including phospholipid metabolism, RNA modification, as well as fatty acid beta-oxidation [118]. As an important gene in the phosphate-transport system, *pstS* is also distributed widely in cyanobacteria, eubacteria, and archaea [120]. Under phosphate stress conditions, the gene *pstS* can modulate phosphate absorption and assimilation and contribute to the pyrolysis cycle in the host cell [21,121]. Both the *phoH* and *pstS* genes might improve phosphorus acquisition during the infection of the host cells.

## 7. AMGs with the Potential to Impact Methylation

Methylation plays a key role in the infection process. In the sequenced cyanophage genomes, DNA methylase (23/153), DNA adenine methylase (55/153), cytosine methyltransferase (11/153), and type II N-methyl DNA methyltransferase (13/153) were found to be involved in methylation. DNA coding and base-pairing functions are not affected by methylation modifications. In prokaryotes, modified bases appear primarily to protect the host from the infection of bacteriophages (and other genomic parasites) [122].

## 8. Biosynthetic Gene Clusters (BGCs): What Is the Function of BGCs in Phage?

Bacteria can produce diverse metabolites, which are a rich source of industrially relevant natural products. The synthetic pathway of these metabolites is encoded by groups of genes called biosynthetic gene clusters (BGCs). BGCs can affect the interaction and competence among microorganisms. Further, BGCs are extremely important for the mining of new bioactivities [123]. In a recent study, the structure and function of phage-encoded BGCs were investigated [123]. pBGCs are rare, and most of them reside within temperate phages, which infect commensal or pathogenic bacterial hosts. The majority of pBGCs were found to encode for bacteriocins [123], which have been shown to provide a clear competitive fitness advantage for the infected bacterium. Through genetic and genomic comparisons, researchers revealed a strong association between the pBGC type and phage host range, which demonstrated bacteriocins are encoded in temperate phages of a few commensal bacterial genera, and lysogenic conversion also provides an evolutionary benefit to the phage itself [123].

In our review, we also investigated the presence of BGCs in the 153 publicly accessible cyanophage genomes using antiSMASH. However, no BGCs in the 153 cyanophage genomes were predicted—an unsurprising revelation due to the documented rarity of pBGCs. A previous study utilized 10,062 high-quality phage genomes, which were available in the PATRIC database, and detected the presence of pBGCs in 69 genomes. Furthermore, 15,184 high-quality bacterial genomes available from NCBI were screened by ProphET to extract prophage regions and were subsequently screened by antiSMASH for the detection of pBGCs. This revealed the presence of only 307 pBGC regions [123]. However, with the increased discovery and genomic sequencing of cyanophages, it is likely that more pBGCs will be discovered. Understanding the natural function and distribution of pBGCs may provide insight into the mechanisms through which cyanophages infect, control, and utilize the host.

## 9. Response of Hosts to Cyanophage Infection

In the previous part of the review, we focused on how cyanophages affected their hosts using the AMGs followed by a discussion of the response of hosts to the infection of cyanophages. After cyanophage infection, most host gene expression is suppressed. However, some host gene expression is enhanced.

In a recent study [124], researchers replaced general medium using medium containing 98 atoms% ^15^NO^3−^ as the sole N source after the S-SM1 phage had infected *Synechococcus* WH8102. They found 12 host-encoded proteins that continued to be produced despite the general suppression of host gene expression. Of these 12 proteins, 8 of them have homologs (i.e., AMGs) in the genomes of S-SM1 or other phages and/or on putative viral genome fragments from aquatic viral metagenome datasets. These eight proteins included heme oxygenase, methionyl aminopeptidase, ribosomal protein S21, glycogen phosphorylase, phosphogluconate dehydrogenase (Gnd), transaldolase (TalC), cysteine synthase A, and the CP12 repressor. Methionyl aminopeptidase removes initiator methionine residues, which is the second step in the N-terminal methionine excision (NME) pathway of protein maturation. NME is necessary for the stable assembly of the D1/D2 reaction center of photosystem II [125], so the continued production of methionyl aminopeptidase may be linked to phage PSII protein expression. Ribosomal protein S21 is necessary for mRNA binding and translation initiation [126], which can enhance viral fitness. We have discussed the other six proteins in the previous review.

The synthesis of the other 4 of the 12 host proteins may point to cellular defense mechanisms against phage infection. The Rho termination factor is involved in the silencing of foreign DNA elements as a defense against the deleterious effects of their expression [127]. Ribonuclease J may act to degrade phage transcripts and inhibit infection progress [124].

This study suggests that phages without some AMGs may also maintain the function of some metabolism pathways, which are necessary for the phages using the hosts’ genes (such as S-SM1). This study may also explain why there are more AMGs in the myocyanophages than in the other two types of cyanophages. It is likely that the production of some of the host gene’s proteins is not enough for myocyanophages growth and may need more energy, thus having more AMGs present than the other two types of cyanophages.

## 10. Conclusions

In the aqueous system, cyanobacteria can be lysed by cyanophages, which, in turn, release dissolved carbon and nutrients. The dissolved organic matter (DOM) plays essential roles in the ocean’s recycling system. By creating sticky lysates, phages can effectively shuttle organic carbon from the surface to the deep ocean. Cyanophages play a vital role in the evolution of cyanobacteria, exerting control over cyanobacterial abundance, population dynamics, and the structure of natural communities. They serve as a global reservoir of genetic information, functioning as vectors for gene transfer and endowing cyanobacteria with new properties that influence the rate and direction of evolutionary processes. Lysogeny markedly contributes to the maintenance of the gene pool and facilitates ecological adaptation among cyanobacteria. The integration of multiple cyanobacterial genes into cyanophage genomes provides evidence of genetic transfer between hosts and phages, which drives adaptive microevolutionary processes. Molecular analyses of cyanophage–host interactions strongly support the concept of coevolution between cyanophages and cyanobacterial genomes [128].

From the 1990s until now, there has been an increase in the number of cyanophages that have been isolated and their genomes sequenced. It is evident that there are many homologous genes between cyanophages and their host, and it is possible for genetic material exchange and cell metabolism to occur after infection. In our review, all publicly accessible cyanophage genomes in NCBI were found, and 45 AMGs, which play a key role in the metabolism process, were identified in these cyanophage genomes. These were evaluated and categorized into five categories. It was shown that there are twelve genes involved in photosynthesis, six genes involved in carbon metabolism, five genes involved in methylation, three genes involved in phosphate metabolism, and 15 regulatory factor genes. After phage infection, the biosynthesis of PBS is enhanced through the expression of photosynthesis family genes from cyanophages. In order to supply more ATP for cyanophage DNA biosynthesis, the expression levels of genes involved in photosynthesis are also enhanced. Cyanophages also redirect carbon flux away from carbon fixation pathways towards dNTP and energy production, which may be used for the generation and assembly of cyanophages. Through the enhancement of phosphate metabolism genes, increased phosphate can be used for augmented DNA synthesis following infection. The regulatory genes in cyanophages can improve the activity and stability of some metabolism pathways. Finally, the methylation-related genes can improve the infection process by preventing host cleavage. Together, these AMGs not only play a beneficial role in the cyanophage during the infection cycle but, on the whole, appear to make a substantial contribution to increasing photosynthesis and global oxygen levels. The ability to enhance an infected host’s biosynthetic capability confers potential advantage to a phage, providing further carbon flux that may be channeled toward phage replication [54,55,129]. From an ecological and biotechnological viewpoint, an enhanced understanding of these infection processes holds the opportunity to use them as non-GM tools for organism engineering, enhancing their usefulness as chemical factories and potentially harnessing them for accelerated carbon capture and oxygen generation. The role of cyanophages in contributing to specialized metabolites, such as the production of halometabolites, is intriguing and will form the core of prominent future research.

## Figures and Tables

**Table 1:** Overview of cyanophage genomes.

Phage	Taxonomy	Host	Geographic Distribution	Genome Length (Kb)	GC%	Protein	Gene	tRNA	Accession
*Anabaena* phage A-4L [12]	Podoviridae	*Anabaena*	Russia	41.75	43.4	38	38		NC_024358
Cyanophage 9515-10a	Podoviridae	*Prochlorococcus*	Sargasso Sea, North Atlantic Ocean	47.06	39.2	55	56		NC_016657
Cyanophage BHS3	Podoviridae		South Africa	10.03	54.9	9			MF098555
Cyanophage KBS-P-1A	Podoviridae	*Synechococcus*	USA	45.73	47.4	57	58		NC_020865
Cyanophage KBS-S-1A	Podoviridae	*Synechococcus*	USA	32.4	44.7	37	42		JF974297
Cyanophage KBS-S-2A [13]	Siphoviridae	*Synechococcus*	Atlantic: Chesapeake Bay	40.66	49	64	64		NC_020854
Cyanophage MED4-117	Siphoviridae			38.83	37.2	63	63		NC_020857
Cyanophage NATL1A-7	Podoviridae	*Prochlorococcus*	Gulf of Aqaba, Red Sea	47.74	38.7	65	69	1	NC_016658
Cyanophage NATL2A-133	Podoviridae		North Pacific Ocean	47.54	39.9	62	66		NC_016659
Cyanophage PP [14]	Podoviridae	*Leptolyngbya foveolarum*	China	42.48	46.4	41	41		NC_022751
Cyanophage P-RSM1	Myoviridae		Gulf of Aqaba, Red Sea	177.21	40.2	212	215	2	NC_021071
Cyanophage P-RSM6	Myoviridae		Red Sea	192.5	39.3	221	224	3	NC_020855
Cyanophage P-SS1	Myoviridae	*Prochlorococcus*	North Atlantic Ocean	178.28	36.7	216	217		JF974306
Cyanophage PSS2 [15]	Siphoviridae	*Prochlorococcus*		10.7	52.3	132	132		NC_013021
Cyanophage P-SSP2	Podoviridae			45.89	37.9	56	59		NC_016656
Cyanophage P-TIM40	Myoviridae	*Prochlorococcus*		188.63	40.7	235	236	1	NC_028663
Cyanophage S-RIM4	Myoviridae	*Synechococcus*	USA: Narragansett Bay; Rhode Island	175.46	41.2	228	237	9	MK493321
Cyanophage S-RIM12 [16]	Myoviridae		USA: Narragansett Bay; Rhode Island	174.3	39.6	215			KX349318
Cyanophage S-RIM14 [17]	Myoviridae		USA	179.5	41	216			KX349306
Cyanophage S-RIM32 [18]	Myoviridae		USA	194.44	39.9	230	240	10	NC_031235
Cyanophage S-RIM50	Myoviridae	*Synechococcus*	USA	174.31	40.3	227	235	8	NC_031242
Cyanophage SS120-1	Podoviridae	*Prochlorococcus*	Sargasso Sea, North Atlantic Ocean	46.99	40.5	52			NC_020872
Cyanophage S-SSM2	Myoviridae	*Synechococcus*	Sargasso Sea	179.98	41	207	208	1	JF974292
Cyanophage S-SSM6a	Myoviridae	*Synechococcus*	Atlantic Ocean	232.88	39	304	310	5	HQ317391
Cyanophage S-SSM6b	Myoviridae	*Synechococcus*	Sargasso Sea, North Atlantic Ocean	182.37	39.4	218	221	3	HQ316603
Cyanophage S-TIM4	Myoviridae		Israel	176.5	37.7	235	235	3	NC_048015
Cyanophage S-TIM5 [10]	Myoviridae	*Synechococcus*	Gulf of Aqaba, Red Sea	161.44	40.5	180	190	10	NC_019516
Cyanophage Syn2	Myoviridae	*Synechococcus*	Sargasso Sea	175.6	41.3	201	209	6	HQ634190
Cyanophage Syn10	Myoviridae	*Synechococcus*	Gulf Stream	177	40.6	205	216	6	HQ634191
Cyanophage Syn30	Myoviridae	*Synechococcus*	NE Providence Channel	178.81	39.9	209	216	6	NC_021072
*Microcystis* phage MACPNOA1	Siphoviridae	*Microcystis*		42.47	68.3	53			KY697807
*Microcystis* virus Ma-LMM01 [19]	Myoviridae	*Microcystis*	Japan	1621	46	184	186	2	NC_008562
*Phormidium* virus WMP3	Podoviridae	*Phormidium*		43.25	46.5	41	41		NC_009551
*Phormidium* virus WMP4 [20]	Podoviridae	*Phormidium*		40.94	51.8	45	45		NC_008367
*Prochlorococcus* phage MED4-184	Siphoviridae	*Prochlorococcus*		38.33	37.2	59	60		NC_020847
*Prochlorococcus* phage MED4-213	Myoviridae	*Prochlorococcus*	North Pacific Ocean	180.98	37.8	216	218		NC_020845
*Prochlorococcus* phage P-GSP1	Podoviridae	*Prochlorococcus*	Gulf Stream, North Atlantic Ocean	44.95	39.6	49	53		NC_020878
*Prochlorococcus* phage P-HM1 [21]	Myoviridae	*Prochlorococcus*		181.04	37.8	241	241		NC_015280
*Prochlorococcus* phage P-HM2 [21]	Myoviridae	*Prochlorococcus*		183.81	38	242	242		NC_015284
*Prochlorococcus* phage P-RSM4 [21]	Myoviridae	*Prochlorococcus*	Red Sea	176.43	37.6	239	242	3	NC_015283
*Prochlorococcus* phage P-RSP2	Podoviridae	*Prochlorococcus*	Gulf of Aqaba, Red Sea	42.26	34	46	48		HQ332139
*Prochlorococcus* phage P-SSM2	Myoviridae	*Prochlorococcus*		252.4	35.5	332			GU071092
*Prochlorococcus* phage P-SSM3	Myoviridae	*Prochlorococcus*	Atlantic Ocean	179.06	36.7	214	223		NC_021559
*Prochlorococcus* phage P-SSM4 [21]	Myoviridae	*Prochlorococcus*	Sargasso Sea, North Atlantic Ocean	178.25	36.7	221	221		NC_006884.2
*Prochlorococcus* phage P-SSM5	Myoviridae	*Prochlorococcus*	Sargasso Sea, North Atlantic Ocean	252.01	35.5	319	331	1	HQ632825
*Prochlorococcus* phage P-SSM7 [21]	Myoviridae	*Prochlorococcus*		1828	37	237	241	4	NC_015290
*Prochlorococcus* phage P-SSP3	Podoviridae	*Prochlorococcus*	Sargasso Sea, North Atlantic Ocean	46.2	37.9	53	56		NC_020874
*Prochlorococcus* phage P-SSP6	Podoviridae	*Prochlorococcus*	Sargasso Sea, North Atlantic Ocean	47.04	39.2	48	54		HQ634152
*Prochlorococcus* phage Syn1 [21]	Myoviridae	*Prochlorococcus*		191.2	40.6	234	240	6	NC_015288
*Prochlorococcus* phage Syn33 [21]	Myoviridae	*Prochlorococcus*	Gulf Stream, North Atlantic Ocean	174.29	39.6	227	232	5	NC_015285
*Prochlorococcus* virus PSSP7	Podoviridae	*Prochlorococcus*		45	38.8	58			NC_006882
*Synechococcus* phage KBS-M-1A	Myoviridae	*Synechococcus*	USA	171.74	40.6	213	221	8	JF974293
*Synechococcus* phage S-B68	Myoviridae	*Synechococcus*	China	163.98	51.7	229			MK016664
*Synechococcus* phage S-CAM1	Myoviridae	*Synechococcus*	USA	197.5	43	250			NC_020837
*Synechococcus* phage S-CAM8	Myoviridae	*Synechococcus*	USA	171.8	39.2	207			NC_021530
*Synechococcus* phage S-CBM2	Myoviridae	*Synechococcus*	USA	180.89	39.7	197	212	8	HQ633061
*Synechococcus* phage S-CBP4	Podoviridae	*Synechococcus*		429.8	44.35	47			NC_048183
*Synechococcus* phage S-CBP42 [22]	Podoviridae	*Synechococcus*		45.2	54.6	51			NC_029031
*Synechococcus* phage S-EIVl [23]	unclassified bacterial viruses	*Synechococcus*	Canada	798	45.9		129		KJ410740
*Synechococcus* phage S-IOM18	Myoviridae	*Synechococcus*	Arabian Sea	171.8	40.6	209	219	7	NC_021536
*Synechococcus* phage S-PM2 [8]	Myoviridae	*Synechococcus*	English Channel	191.5	37.8	227			NC_006820
*Synechococcus* phage S-RIM2 [17]	Myoviridae	*Synechococcus*	USA	175.3	42.2	214			KX349284
*Synechococcus* phage S-RIM8 [17]	Myoviridae	*Synechococcus*	USA	170.3	40.6	221			NC_047733
*Synechococcus* phage S-RIP1	Podoviridae	*Synechococcus*	USA	44.89	42.9	55	61		NC_020867
*Synechococcus* phage S-RIP2	Podoviridae	*Synechococcus*	USA	45.73	47.3	54	57		NC_020838
*Synechococcus* phage S-RSM4 [24]	Myoviridae	*Synechococcus*	Gulf of Aqaba, Red Sea	194.45	41	237	249	12	NC_013085
*Synechococcus* phage S-ShM2 [21]	Myoviridae	*Synechococcus*		179.56	41	230	231	1	NC_015281
*Synechococcus* phage S-SKS1	Myoviridae	*Synechococcus*	North Atlantic Ocean	208.01	36	281	302	11	NC_020851
*Synechococcus* phage S-SM1 [21]	Myoviridae	*Synechococcus*		174.08	41	234	240	6	NC_015282
*Synechococcus* phage S-SM2 [21]	Myoviridae	*Synechococcus*		190.79	40.4	267	278	11	NC_015279
*Synechococcus* phage S-SSM4	Myoviridae	*Synechococcus*	W Sargasso Sea	182.8	39.4	220	223	3	NC_020875
*Synechococcus* phage S-SSM5 [21]	Myoviridae	*Synechococcus*	Sargasso Sea	1768	40	225	229	4	NC_015289
*Synechococcus* phage S-SSM7 [21]	Myoviridae	*Synechococcus*	Sargasso Sea	232.88	39	319	324	5	NC_015287
*Synechococcus* phage S-WAM1 [18]	Myoviridae	*Synechococcus*	USA	185	44.7	221	225	4	NC_031944
*Synechococcus* phage S-WAM2 [18]	Myoviridae	*Synechococcus*	USA	186.39	41.3	229	241	12	NC_031935
*Synechococcus* phage syn9 [25]	Myoviridae	*Synechococcus*	North Atlantic Ocean	177.3	40.6	226	232	6	NC_008296.2
*Synechococcus* phage Syn19 [21]	Myoviridae	*Synechococcus*	Sargasso Sea, North Atlantic Ocean	175.23	41.3	215	221	6	NC_015286
*Plectonema* phage JingP1	unclassified bacterial viruses	*Plectonema*	China	40.8	52.3	49			ON677538
Cyanophage Pf-WMP3 [26]	Podoviridae	*Phormidium*		43.25	46.5	41	41		NC_009551
Cyanophage Pf-WMP4 [20]	Podoviridae	*Phormidium*		40.94	51.8	45	45		NC_008367
*Synechococcus* phage P60 [27]	Podoviridae	*Synechococcus*		46.68	53.3	55	55		NC_003390
*Microcystis* cyanophage Mwe-Yong1112-1	Siphoviridae	*Microcystis*		39.68	66.6	53			MZ436628
*Microcystis* cyanophage vB_MaeS-yong1	Siphoviridae	*Microcystis*	China	43.67	66.7	56			MT855965
Cyanophage P-RSM3	Myoviridae	*Prochlorococcus*	Gulf of Aqaba, Red Sea	178.75	36.7	208	211	3	HQ634176
Cyanophage S-2L	Siphoviridae			45.087	69.25	56			MW334946
*Synechococcus* phage MRHenn-2013a	Podoviridae	*Synechococcus*	Chesapeake Bay	92.47	51.2	130	131	1	JF974303
*Synechococcus* phage S-SRP02 [28]	Podoviridae	*Synechococcus*	Singapore	424	63.4	47	47		MW822601
*Synechococcus* phage S-SRP01	Podoviridae	*Synechococcus*	Singapore	45.02	48.9	56			MW015080
*Synechococcus* phage S-SBP1	Podoviridae	*Synechococcus*	China	45.52	46.8	55	55		MT424636
*Synechococcus* phage S-B28	Podoviridae	*Synechococcus*	China	45.42	42	55	55		NC_048171
*Synechococcus* phage S-CBP1 [22]	Podoviridae	*Synechococcus*		46.55	47.6	50	51		NC_025456
*Synechococcus* phage S-CBP2 [22]	Podoviridae	*Synechococcus*		46.24	55	53	53		NC_025455
*Synechococcus* phage S-CBP3	Podoviridae	*Synechococcus*		46.62	46.85	54			NC_025461
*Synechococcus* phage S-CRM01 [29]	Myoviridae	*Synechococcus*	USA	178.56	39.7	297	297	33	NC_015569
*Synechococcus* phage BUCT-ZZ01	Myoviridae	*Synechococcus*	China	156.06	38.5	236			ON615601
*Synechococcus* phage S-H38	Myoviridae	*Synechococcus*	China	180.22	42.4	211		8	NC_070964
*Synechococcus* phage S-SRM01	Myoviridae	*Synechococcus*	Singapore	240.84	35.6	353			NC_070963
*Synechococcus* phage S-N03	Myoviridae	*Synechococcus*	China	167.07	50	247		1	NC_070959
*Synechococcus* phage S-H34	Myoviridae	*Synechococcus*	China	167.04	50	246		5	NC_070965
*Synechococcus* phage B3 [30]	Myoviridae	*Synechococcus*	Canada	244.93	35.4	370	391	21	MN695334
*Synechococcus* phage B23 [30]	Myoviridae	*Synechococcus*	Canada	243.63	35.4	365	384	19	MN695335
*Synechococcus* phage S-SCSM1	Myoviridae	*Synechococcus*	China	228.83	36.8	282	293	9	NC_070962
*Synechococcus* phage S-H68	Myoviridae	*Synechococcus*	China	79.64	40.4	117			MK016663
Synechoccus phage S-B43	Myoviridae	*Synechococcus*	China	214	39.4	262			MN018232
*Synechococcus* phage S-B05	Myoviridae	*Synechococcus*	China	209	39.9	268			MK799832
*Synechococcus* phage S-SZBM1	Myoviridae;	*Synechococcus*	China	177.83	43.3	218	218	6	NC_070839
*Synechococcus* phage ACG-2014f	Myoviridae;	*Synechococcus*		222.3	41.6	286			NC_026927
*Synechococcus* phage S-H9-1	Myoviridae;	*Synechococcus*	China	192.45	41	229		12	NC_070961
*Synechococcus* phage S-H9-2	Myoviridae;	*Synechococcus*	China	187.32	40.3	202		14	NC_070960
*Synechococcus* phage SynMITS9220M01	Myoviridae;	*Synechococcus*	Pacific Ocean	190.24	38.8	245	255	10	MT408532
*Synechococcus* phage S-H25	Myoviridae;	*Synechococcus*	China	190.24	38.8	245	255	10	MT162468
*Synechococcus* phage S-P4	Myoviridae;	*Synechococcus*	China	158.48	39.9	195	195		NC_048102
Synechoccus phage S-E7	Myoviridae;	*Synechococcus*	China	177.62	39.9	216			MH920640
*Synechococcus* phage S-B64	Myoviridae;	*Synechococcus*	China	151.87	41.3	156	156		NC_055744
*Synechococcus* phage Bellamy	Myoviridae;	*Synechococcus*	USA	204.93	41	269	279	10	NC_047838
*Synechococcus* phage S-H35	Myoviridae;	*Synechococcus*	China	174.23	41.2	204	204		NC_055719
*Synechococcus* phage S-CAM9 [18]	Myoviridae;	*Synechococcus*	USA	174.8	39	228			NC_031922
*Synechococcus* phage S-CAM7 [18]	Myoviridae;	*Synechococcus*	USA	215.2	41.2	266			NC_031927
*Synechococcus* phage S-CAM4 [18]	Myoviridae;	*Synechococcus*	USA	192	38.6	238			NC_031900
*Synechococcus* phage S-CAM3 [18]	Myoviridae;	*Synechococcus*	USA	197.8	41.6	239			NC_031906
*Synechococcus* phage S-CAM22 [18]	Myoviridae;	*Synechococcus*	USA; Mexico	172.3	39.9	214			NC_055710
*Synechococcus* cyanophage S-WAM2	Myoviridae;	*Synechococcus*	USA	186.39	41.3	229	241	12	NC_031935
*Synechococcus* cyanophage S-WAM1	Myoviridae;	*Synechococcus*	USA	185	44.7	221	225	4	NC_031944
*Synechococcus* phage ACG-2014j	Myoviridae;	*Synechococcus*		192	38.65	226			NC_062735
*Synechococcus* phage ACG-2014i	Myoviridae	*Synechococcus*		190.77	39	212	212		NC_027132
*Synechococcus* phage metaG-MbCM1	Myoviridae	*Synechococcus*		172.88	39.8	234	234		NC_019443
*Synechococcus* phage ACG-2014g	Myoviridae	*Synechococcus*		174.89	39.3	216	216		NC_026924
*Synechococcus* phage ACG-2014e	Myoviridae	*Synechococcus*		189.4	38.9	214			NC_062736
*Synechococcus* phage ACG-2014d	Myoviridae	*Synechococcus*		179.2	40.3	218			NC_062734
*Synechococcus* phage ACG-2014b	Myoviridae	*Synechococcus*		172.7	39	216			NC_047719
*Synechococcus* phage ACG-2014a	Myoviridae	*Synechococcus*		171.3	39.4	208			KJ019163
*Synechococcus* phage S-MbCM100 [31]	Myoviridae	*Synechococcus*	USA	170.44	39.4	210	210		NC_023584
*Synechococcus* phage S-MbCM25 [31]	Myoviridae	*Synechococcus*	USA	176.04	39	215	215		KF156339
*Synechococcus* phage S-MbCM7 [31]	Myoviridae	*Synechococcus*	USA	189.31	40.5	221	221		NC_023587
*Synechococcus* phage S-RIM8 A.HR5 [32]	Myoviridae	*Synechococcus*	North Atlantic Ocean	168.33	40.6	203	211	8	HQ317385
*Synechococcus* phage S-RIM8 A.HR3 [32]	Myoviridae	*Synechococcus*	North Atlantic Ocean	171.21	40.6	212	220	8	JF974289
*Synechococcus* phage S-RIM8 A.HR1 [32]	Myoviridae	*Synechococcus*	North Atlantic Ocean	171.21	40.6	211	219	8	NC_020486
*Synechococcus* phage S-RIM2 R9_2006	Myoviridae	*Synechococcus*	North Atlantic Ocean	175.42	42.2	210	217	6	HQ317291
*Synechococcus* phage S-RIM2 R1_1999	Myoviridae	*Synechococcus*	North Atlantic Ocean	175.43	42.2	210	216	6	NC_020859
*Synechococcus* phage S-RIM2 R21_2007	Myoviridae	*Synechococcus*	North Atlantic Ocean	175.43	42.2	208	214	6	HQ317290
*Synechococcus* phage Syn30	Myoviridae	*Synechococcus*	NE Providence Channel	178.81	39.9	209	216	6	NC_021072
*Synechococcus* phage ACG-2014c	Myoviridae	*Synechococcus*		176	39	215			NC_019444
*Synechococcus* phage ACG-2014h	Myoviridae	*Synechococcus*	USA	189.31	40.5	221	221		NC_023587
*Synechococcus* phage S-H1	Siphoviridae	*Synechococcus*	China	396	53.7	64			MK105819
*Synechococcus* phage S-LBS1	Siphoviridae	*Synechococcus*	France	34.64	60.2	52	52		MG271909
*Synechococcus* phage S-CBS1 [9]	Siphoviridae	*Synechococcus*	USA	30.33	58.7	43	43		NC_016164
*Synechococcus* phage S-CBS4 [9]	Siphoviridae	*Synechococcus*		69.4	50.8	105			NC_016766
*Synechococcus* phage S-CBS3 [9]	Siphoviridae	*Synechococcus*		33	60.7	46	46		NC_015465
*Synechococcus* phage S-CBS2 [9]	Siphoviridae	*Synechococcus*		72.33	54.5	102	102		NC_015463
*Synechococcus* phage S-T4	Myoviridae	*Synechococcus*	China	181.08	38.9	226	226		NC_048049
*Synechococcus* phage S-CBWM1 [33]	Myoviridae	*Synechococcus*	USA	139.07	51.6	178	216	36	NC_048106
*Synechococcus* virus S-ESS1	Siphoviridae	*Synechococcus*	China	60.36	60.9	52	52		NC_052968
*Synechococcus* virus Syn5 [34]	Podoviridae	*Synechococcus*		46.21	55	61	61		NC_009531

**Table 2:** AMGs in the sequenced cyanophage genomes.

Protein Function	Protein	Ratios	Protein Function	Protein	Ratios
Photosynthesis	photosystem II reaction center protein PsbA/D1	111/153	Methylation	DNA methylase	23/153
	photosystem II D2 protein	77/153		DNA adenine methylase Dam	55/153
	phycocyanobilin:ferredoxin oxidoreductase (PcyA)	16/153		cytosine methyltransferase	11/153
	ferredoxin (PetF)	44/153		type II N-methyl DNA methyltransferase (group alpha)	13/153
	phycobilin lyase (CpeT)	31/153		phage host specificity protein	3/153
	phycoerythrobilin synthase (PebS)	9/153			
	phycoerythrobilin	4/153	Phosphate metabolism	phosphate-starvation-inducible protein (PhoH)	101/153
	plastoquinol terminal oxidase PtoX	35/153		phosphate-induced stress protein	2/153
	plastocyanin (PetE)	70/153		phosphate ABC transporter phosphate-binding protein PstS	31/153
	heme oxygenase (Ho)	8/153			
	hli gene family/high-light inducible protein	90/153			
	thioredoxin	5/153	Regulatory factor	CobS/cobO/cobA	39/153
	rfaE bifunctional protein	1/153		RNA polymerase sigma factor RpoD/RpoS	65/153
	2OG-Fe(II) oxygenase family oxidoreductase	76/153		ERF family protein	1/153
	NADH dehydrogenase subunit I	1/153		MazG	64/153
	NAD synthetase	24/153		RegA photosynthetic apparatus regulatory protein	48/153
Carbon metabolism	carboxylesterase	24/153		transcriptional regulator	7/153
	FAD-dependent thymidylate synthase/thymidylate synthase	95/153		late promoter transcriptional accessory protein	41/153
	transaldolase family protein TalC in this family	96/153		CP12 carbon metabolic regulator	31/153
	glucose 6-phosphate dehydrogenase Zwf	25/153		MarR family protein	3/153
	6-phosphogluconate dehydrogenase Gnd	22/153		translational regulator	3/153
	UDP-glucose 6-dehydrogenase	1/153		XRE family transcriptional regulator	1/153
	acyl carrier protein ACP	2/153		Nrd	48/153
	fructose-6-phosphate aldolase	1/153		tryptophan halogenase	20/153

## Data Availability

All data analysed in this study is obtained from the NCBI database. https://www.ncbi.nlm.nih.gov/, accessed on 30 May 2023.

## References

[1] Pomeroy L.R., LeB Williams P.J., Azam F., Hobbie J.E. (2007). The microbial loop. Oceanography.

[2] Wommack K.E., Colwell R.R. (2000). Virioplankton: Viruses in aquatic ecosystems. Microbiol. Mol. Biol. Rev..

[3] Breitbart M., Bonnain C., Malki K., Sawaya N.A. (2018). Phage puppet masters of the marine microbial realm. Nat. Microbiol..

[4] Azam F., Fenchel T., Field J.G., Gray J., Meyer-Reil L., Thingstad F. (1983). The ecological role of water-column microbes in the sea. Mar. Ecol. Prog. Ser..

[5] Moran M.A., Kujawinski E.B., Schroer W.F., Amin S.A., Bates N.R., Bertrand E.M., Braakman R., Brown C.T., Covert M.W., Doney S.C. (2022). Microbial metabolites in the marine carbon cycle. Nat. Microbiol..

[6] Moran M.A., Ferrer-González F.X., Fu H., Nowinski B., Olofsson M., Powers M.A., Schreier J.E., Schroer W.F., Smith C.B., Uchimiya M. (2022). The Ocean’s labile DOC supply chain. Limnol. Oceanogr..

[7] Wilhelm S.W., Suttle C.A. (1999). Viruses and nutrient cycles in the sea: Viruses play critical roles in the structure and function of aquatic food webs. Bioscience.

[8] Mann N.H., Clokie M.R., Millard A., Cook A., Wilson W.H., Wheatley P.J., Letarov A., Krisch H. (2005). The genome of S-PM2, a “photosynthetic” T4-type bacteriophage that infects marine *Synechococcus* strains. J. Bacteriol..

[9] Huang S., Wang K., Jiao N., Chen F. (2012). Genome sequences of siphoviruses infecting marine *Synechococcus* unveil a diverse cyanophage group and extensive phage–host genetic exchanges. Environ. Microbiol..

[10] Sabehi G., Shaulov L., Silver D.H., Yanai I., Harel A., Lindell D. (2012). A novel lineage of myoviruses infecting cyanobacteria is widespread in the oceans. Proc. Natl. Acad. Sci. USA.

[11] Labrie S., Frois-Moniz K., Osburne M., Kelly L., Roggensack S., Sullivan M., Gearin G., Zeng Q., Fitzgerald M., Henn M. (2013). Genomes of marine cyanopodoviruses reveal multiple origins of diversity. Environ. Microbiol..

[12] Ou T., Liao X.-Y., Gao X.-C., Xu X.-D., Zhang Q.-Y. (2015). Unraveling the genome structure of cyanobacterial podovirus A-4L with long direct terminal repeats. Virus Res..

[13] Broadbent J.R., Hughes J.E., Welker D.L., Tompkins T.A., Steele J.L. (2013). Complete genome sequence for Lactobacillus helveticus CNRZ 32, an industrial cheese starter and cheese flavor adjunct. Genome Announc..

[14] Zhou Y., Lin J., Li N., Hu Z., Deng F. (2013). Characterization and genomic analysis of a plaque purified strain of cyanophage PP. Virol. Sin..

[15] Sullivan M.B., Krastins B., Hughes J.L., Kelly L., Chase M., Sarracino D., Chisholm S.W. (2009). The genome and structural proteome of an ocean siphovirus: A new window into the cyanobacterial ‘mobilome’. Environ. Microbiol..

[16] Marston M.F., Taylor S., Sme N., Parsons R.J., Noyes T.J., Martiny J.B. (2013). Marine cyanophages exhibit local and regional biogeography. Environ. Microbiol..

[17] Marston M.F., Martiny J.B. (2016). Genomic diversification of marine cyanophages into stable ecotypes. Environ. Microbiol..

[18] Crummett L.T., Puxty R.J., Weihe C., Marston M.F., Martiny J.B. (2016). The genomic content and context of auxiliary metabolic genes in marine cyanomyoviruses. Virology.

[19] Yoshida T., Nagasaki K., Takashima Y., Shirai Y., Tomaru Y., Takao Y., Sakamoto S., Hiroishi S., Ogata H. (2008). Ma-LMM01 infecting toxic Microcystis aeruginosa illuminates diverse cyanophage genome strategies. J. Bacteriol..

[20] Liu X., Shi M., Kong S., Gao Y., An C. (2007). Cyanophage Pf-WMP4, a T7-like phage infecting the freshwater cyanobacterium Phormidium foveolarum: Complete genome sequence and DNA translocation. Virology.

[21] Sullivan M.B., Huang K.H., Ignacio-Espinoza J.C., Berlin A.M., Kelly L., Weigele P.R., Defrancesco A.S., Kern S.E., Thompson L.R., Young S. (2010). Genomic analysis of oceanic cyanobacterial myoviruses compared with T4-like myoviruses from diverse hosts and environments. Environ. Microbiol..

[22] Huang S., Zhang S., Jiao N., Chen F. (2015). Comparative genomic and phylogenomic analyses reveal a conserved core genome shared by estuarine and oceanic cyanopodoviruses. PLoS ONE.

[23] Chénard C., Chan A., Vincent W., Suttle C. (2015). Polar freshwater cyanophage S-EIV1 represents a new widespread evolutionary lineage of phages. ISME J..

[24] Millard A.D., Zwirglmaier K., Downey M.J., Mann N.H., Scanlan D.J. (2009). Comparative genomics of marine cyanomyoviruses reveals the widespread occurrence of *Synechococcus* host genes localized to a hyperplastic region: Implications for mechanisms of cyanophage evolution. Environ. Microbiol..

[25] Weigele P.R., Pope W.H., Pedulla M.L., Houtz J.M., Smith A.L., Conway J.F., King J., Hatfull G.F., Lawrence J.G., Hendrix R.W. (2010). Genomic and structural analysis of Syn9, a cyanophage infecting marine *Prochlorococcus* and *Synechococcus*. Environ. Microbiol..

[26] Liu X., Kong S., Shi M., Fu L., Gao Y., An C. (2008). Genomic analysis of freshwater cyanophage Pf-WMP3 infecting cyanobacterium Phormidium foveolarum: The conserved elements for a phage. Microb. Ecol..

[27] Chen F., Lu J. (2002). Genomic sequence and evolution of marine cyanophage P60: A new insight on lytic and lysogenic phages. Appl. Environ. Microbiol..

[28] Zhang D., He Y., Gin K.Y.-H. (2022). Genomic characterization of a novel freshwater cyanophage reveals a new lineage of cyanopodovirus. Front. Microbiol..

[29] Dreher T.W., Brown N., Bozarth C.S., Schwartz A.D., Riscoe E., Thrash C., Bennett S.E., Tzeng S.C., Maier C.S. (2011). A freshwater cyanophage whose genome indicates close relationships to photosynthetic marine cyanomyophages. Environ. Microbiol..

[30] Lévesque A.V., Thaler M., Labrie S.J., Marois C., Vincent A.T., Lapointe A.-M., Culley A. (2020). Complete genome sequences for two Myoviridae strains infecting cyanobacteria in a subarctic lake. Microbiol. Resour. Announc..

[31] Deng L., Ignacio-Espinoza J.C., Gregory A.C., Poulos B.T., Weitz J.S., Hugenholtz P., Sullivan M.B. (2014). Viral tagging reveals discrete populations in *Synechococcus* viral genome sequence space. Nature.

[32] Marston M.F., Pierciey F.J., Shepard A., Gearin G., Qi J., Yandava C., Schuster S.C., Henn M.R., Martiny J.B. (2012). Rapid diversification of coevolving marine *Synechococcus* and a virus. Proc. Natl. Acad. Sci. USA.

[33] Xu Y., Zhang R., Wang N., Cai L., Tong Y., Sun Q., Chen F., Jiao N. (2018). Novel phage–host interactions and evolution as revealed by a cyanomyovirus isolated from an estuarine environment. Environ. Microbiol..

[34] Pope W.H., Weigele P.R., Chang J., Pedulla M.L., Ford M.E., Houtz J.M., Jiang W., Chiu W., Hatfull G.F., Hendrix R.W. (2007). Genome sequence, structural proteins, and capsid organization of the cyanophage Syn5: A “horned” bacteriophage of marine *Synechococcus*. J. Mol. Biol..

[35] Bailly-Bechet M., Vergassola M., Rocha E. (2007). Causes for the intriguing presence of tRNAs in phages. Genome Res..

[36] Enav H., Béja O., Mandel-Gutfreund Y. (2012). Cyanophage tRNAs may have a role in cross-infectivity of oceanic *Prochlorococcus* and *Synechococcus* hosts. ISME J..

[37] Dekel-Bird N.P., Sabehi G., Mosevitzky B., Lindell D. (2015). Host-dependent differences in abundance, composition and host range of cyanophages from the Red Sea. Environ. Microbiol..

[38] Puxty R.J., Millard A.D., Evans D.J., Scanlan D.J. (2015). Shedding new light on viral photosynthesis. Photosynth. Res..

[39] Bryant D.A., Frigaard N.-U. (2006). Prokaryotic photosynthesis and phototrophy illuminated. Trends Microbiol..

[40] Chang L., Liu X., Li Y., Liu C.-C., Yang F., Zhao J., Sui S.-F. (2015). Structural organization of an intact phycobilisome and its association with photosystem II. Cell Res..

[41] Yi Z.-W., Huang H., Kuang T.-Y., Sui S.-F. (2005). Three-dimensional architecture of phycobilisomes from Nostoc flagelliforme revealed by single particle electron microscopy. FEBS Lett..

[42] MacColl R. (1998). Cyanobacterial phycobilisomes. J. Struct. Biol..

[43] Gao X., Sun T., Pei G., Chen L., Zhang W. (2016). Cyanobacterial chassis engineering for enhancing production of biofuels and chemicals. Appl. Microbiol. Biotechnol..

[44] Alvey R.M., Biswas A., Schluchter W.M., Bryant D.A. (2011). Effects of modified phycobilin biosynthesis in the cyanobacterium *Synechococcus* sp. strain PCC 7002. J. Bacteriol..

[45] Sane P., Ivanov A., Öquist G., Hüner N. (2012). Photosynthesis: Plastid Biology, Energy Conversion and Carbon Assimilation. Advances in Photosynthesis and Respiration Series.

[46] Thompson L.R., Zeng Q., Chisholm S.W. (2016). Gene Expression Patterns during Light and Dark Infection of *Prochlorococcus* by Cyanophage. PLoS ONE.

[47] Frankenberg-Dinkel N. (2004). Bacterial heme oxygenases. Antioxid. Redox Signal..

[48] Ledermann B., Beja O., Frankenberg-Dinkel N. (2016). New biosynthetic pathway for pink pigments from uncultured oceanic viruses. Environ. Microbiol..

[49] Dammeyer T., Michaelsen K., Frankenberg-Dinkel N. (2007). Biosynthesis of open-chain tetrapyrroles in *Prochlorococcus marinus*. FEMS Microbiol. Lett..

[50] Frankenberg N., Lagarias J.C. (2003). Phycocyanobilin: Ferredoxin Oxidoreductase of *Anabaena* sp. PCC 7120 biochemical and spectroscopic characterization. J. Biol. Chem..

[51] Tu S.-L., Rockwell N.C., Lagarias J.C., Fisher A.J. (2007). Insight into the Radical Mechanism of Phycocyanobilin-Ferredoxin Oxidoreductase (PcyA) Revealed by X-ray Crystallography and Biochemical Measurements. Biochemistry.

[52] Dammeyer T., Hofmann E., Frankenberg-Dinkel N. (2008). Phycoerythrobilin synthase (PebS) of a marine virus. Crystal structures of the biliverdin complex and the substrate-free form. J. Biol. Chem..

[53] Zhao K.-H., Zhang J., Tu J.-M., Böhm S., Plöscher M., Eichacker L., Bubenzer C., Scheer H., Wang X., Zhou M. (2007). Lyase activities of CpcS-and CpcT-like proteins from *Nostoc* PCC7120 and sequential reconstitution of binding sites of phycoerythrocyanin and phycocyanin β-subunits. J. Biol. Chem..

[54] Shan J., Jia Y., Clokie M.R., Mann N.H. (2008). Infection by the ‘photosynthetic’ phage S-PM2 induces increased synthesis of phycoerythrin in *Synechococcus* sp. WH7803. FEMS Microbiol. Lett..

[55] Kamiya N., Shen J.-R. (2003). Crystal structure of oxygen-evolving photosystem II from *Thermosynechococcus* vulcanus at 3.7-Å resolution. Proc. Natl. Acad. Sci. USA.

[56] Melis A. (1999). Photosystem-II damage and repair cycle in chloroplasts: What modulates the rate of photodamage in vivo?. Trends Plant Sci..

[57] Bailey S., Clokie M.R., Millard A., Mann N.H. (2004). Cyanophage infection and photoinhibition in marine cyanobacteria. Res. Microbiol..

[58] Bailey S., Thompson E., Nixon P.J., Horton P., Mullineaux C.W., Robinson C., Mann N.H. (2002). A critical role for the Var2 FtsH homologue of *Arabidopsis thaliana* in the photosystem II repair cycle in vivo. J. Biol. Chem..

[59] Silva P., Thompson E., Bailey S., Kruse O., Mullineaux C.W., Robinson C., Mann N.H., Nixon P.J. (2003). FtsH is involved in the early stages of repair of photosystem II in *Synechocystis* sp. PCC 6803. Plant Cell.

[60] Lindell D., Sullivan M.B., Johnson Z.I., Tolonen A.C., Rohwer F., Chisholm S.W. (2004). Transfer of photosynthesis genes to and from *Prochlorococcus* viruses. Proc. Natl. Acad. Sci. USA.

[61] Lindell D., Jaffe J.D., Johnson Z.I., Church G.M., Chisholm S.W. (2005). Photosynthesis genes in marine viruses yield proteins during host infection. Nature.

[62] Lockau W. (1981). Evidence for a dual role of cytochrome c-553 and plastocyanin in photosynthesis and respiration of the cyanobacterium, *Anabaena variabilis*. Arch. Microbiol..

[63] Thompson L.R., Zeng Q., Kelly L., Huang K.H., Singer A.U., Stubbe J., Chisholm S.W. (2011). Phage auxiliary metabolic genes and the redirection of cyanobacterial host carbon metabolism. Proc. Natl. Acad. Sci. USA.

[64] Battchikova N., Eisenhut M., Aro E.-M. (2011). Cyanobacterial NDH-1 complexes: Novel insights and remaining puzzles. Biochim. Et Biophys. Acta (BBA)-Bioenerg..

[65] Alperovitch-Lavy A., Sharon I., Rohwer F., Aro E.M., Glaser F., Milo R., Nelson N., Béjà O. (2011). Reconstructing a puzzle: Existence of cyanophages containing both photosystem-I and photosystem-II gene suites inferred from oceanic metagenomic datasets. Environ. Microbiol..

[66] Sharon I., Battchikova N., Aro E.-M., Giglione C., Meinnel T., Glaser F., Pinter R.Y., Breitbart M., Rohwer F., Béja O. (2011). Comparative metagenomics of microbial traits within oceanic viral communities. ISME J..

[67] Nowaczyk M.M., Wulfhorst H., Ryan C.M., Souda P., Zhang H., Cramer W.A., Whitelegge J.P. (2011). NdhP and NdhQ: Two novel small subunits of the cyanobacterial NDH-1 complex. Biochemistry.

[68] Appel J., Schulz R. (1996). Sequence analysis of an operon of a NAD (P)-reducing nickel hydrogenase from the cyanobacterium *Synechocystis* sp. PCC 6803 gives additional evidence for direct coupling of the enzyme to NAD (P) H-dehydrogenase (complex 1). Biochim. Biophys. Acta (BBA)-Protein Struct. Mol. Enzymol..

[69] Guedeney G., Corneille S., Cuiné S., Peltier G. (1996). Evidence for an association of ndh B, ndh J gene products and ferredoxin-NADP-reductase as components of a chloroplastic NAD (P) H dehydrogenase complex. FEBS Lett..

[70] Frankenberg N., Mukougawa K., Kohchi T., Lagarias J.C. (2001). Functional genomic analysis of the HY2 family of ferredoxin-dependent bilin reductases from oxygenic photosynthetic organisms. Plant Cell.

[71] Dammeyer T., Frankenberg-Dinkel N. (2008). Function and distribution of bilin biosynthesis enzymes in photosynthetic organisms. Photochem. Photobiol. Sci..

[72] Bailey S., Melis A., Mackey K.R., Cardol P., Finazzi G., van Dijken G., Berg G.M., Arrigo K., Shrager J., Grossman A. (2008). Alternative photosynthetic electron flow to oxygen in marine Synechococcus. Biochim. Et Biophys. Acta (BBA)-Bioenerg..

[73] Mackey K.R., Paytan A., Grossman A.R., Bailey S. (2008). A photosynthetic strategy for coping in a high-light, low-nutrient environment. Limnol. Oceanogr..

[74] He Q., Dolganov N., Björkman O., Grossman A.R. (2001). The High Light-inducible Polypeptides in *Synechocystis* PCC6803 expression and function in high light. J. Biol. Chem..

[75] Havaux M., Guedeney G., He Q., Grossman A.R. (2003). Elimination of high-light-inducible polypeptides related to eukaryotic chlorophyll a/b-binding proteins results in aberrant photoacclimation in *Synechocystis* PCC6803. Biochim. Biophys. Acta (BBA)-Bioenerg..

[76] Promnares K., Komenda J., Bumba L., Nebesarova J., Vacha F., Tichy M. (2006). Cyanobacterial small chlorophyll-binding protein ScpD (HliB) is located on the periphery of photosystem II in the vicinity of PsbH and CP47 subunits. J. Biol. Chem..

[77] Wang Q., Jantaro S., Lu B., Majeed W., Bailey M., He Q. (2008). The High Light-Inducible Polypeptides Stabilize Trimeric Photosystem I Complex under High Light Conditions in *Synechocystis* PCC 6803. Plant Physiol..

[78] Xu H., Vavilin D., Funk C., Vermaas W. (2002). Small Cab-like proteins regulating tetrapyrrole biosynthesis in the cyanobacterium *Synechocystis* sp. PCC 6803. Plant Mol. Biol..

[79] Vavilin D., Yao D., Vermaas W. (2007). Small Cab-like proteins retard degradation of photosystem II-associated chlorophyll in *Synechocystis* sp. PCC 6803: Kinetic analysis of pigment labeling with 15N and 13C. J. Biol. Chem..

[80] Falkowski P.G., Raven J.A. (2013). Aquatic Photosynthesis.

[81] Tamoi M., Miyazaki T., Fukamizo T., Shigeoka S. (2010). The Calvin cycle in cyanobacteria is regulated by CP12 via the NAD(H)/NADP(H) ratio under light/dark conditions. Plant J..

[82] Zinser E.R., Lindell D., Johnson Z.I., Futschik M.E., Steglich C., Coleman M.L., Wright M.A., Rector T., Steen R., McNulty N. (2009). Choreography of the transcriptome, photophysiology, and cell cycle of a minimal photoautotroph, prochlorococcus. PLoS ONE.

[83] Thorell S., Schürmann M., Sprenger G.A., Schneider G. (2002). Crystal structure of decameric fructose-6-phosphate aldolase from *Escherichia coli* reveals inter-subunit helix swapping as a structural basis for assembly differences in the transaldolase family. J. Mol. Biol..

[84] Schneider S., Sandalova T., Schneider G., Sprenger G.A., Samland A.K. (2008). Replacement of a Phenylalanine by a Tyrosine in the Active Site Confers Fructose-6-phosphate Aldolase Activity to the Transaldolase of *Escherichia coli* and Human Origin. J. Biol. Chem..

[85] Puxty R.J., Millard A.D., Evans D.J., Scanlan D.J. (2016). Viruses Inhibit CO_2_ Fixation in the Most Abundant Phototrophs on Earth. Curr. Biol..

[86] Hurwitz B.L., Hallam S.J., Sullivan M.B. (2013). Metabolic reprogramming by viruses in the sunlit and dark ocean. Genome Biol..

[87] Lorenz M.C., Fink G.R. (2002). Life and Death in a Macrophage: Role of the Glyoxylate Cycle in Virulence. Eukaryot. Cell.

[88] Markovitz A., Sydiskis R.J., Lieberman M.M. (1967). Genetic and Biochemical Studies on Mannose-Negative Mutants That Are Deficient in Phosphomannose Isomerase in *Escherichia coli* K-12. J. Bacteriol..

[89] Kholssi R., Lougraimzi H. (2023). Effects of global environmental change on microalgal photosynthesis, growth and their distribution. Mar. Environ. Res..

[90] Paerl H.W., Otten T.G. (2013). Harmful cyanobacterial blooms: Causes, consequences, and controls. Microb. Ecol..

[91] Howard-Varona C., Lindback M.M., Bastien G.E., Solonenko N., Zayed A.A., Jang H., Andreopoulos B., Brewer H.M., del Rio T.G., Adkins J.N. (2020). Phage-specific metabolic reprogramming of virocells. ISME J..

[92] Gyaneshwar P., Paliy O., Mcauliffe J., Popham D.L., Jordan M.I., Kustu S. (2005). Sulfur and Nitrogen Limitation in *Escherichia coli* K-12: Specific Homeostatic Responses. J. Bacteriol..

[93] Guédon E., Martin-Verstraete I. (2006). Cysteine Metabolism and Its Regulation in Bacteria.

[94] Ensign S.A. (2010). Revisiting the glyoxylate cycle: Alternate pathways for microbial acetate assimilation. Mol. Microbiol..

[95] Dolan S.K., Welch M. (2018). The Glyoxylate Shunt, 60 Years on. Annu. Rev. Microbiol..

[96] Ingalls A., Shah S., Hansman R., Aluwihare L., Santos G., Druffel E., Pearson A. (2006). Quantifying archaeal community autotrophy in the mesopelagic ocean using natural radiocarbon. Proc. Natl. Acad. Sci. USA.

[97] Sawa N., Tatsuke T., Ogawa A., Hirokawa Y., Osanai T., Hanai T. (2019). Modification of carbon metabolism in *Synechococcus* elongatus PCC 7942 by cyanophage-derived sigma factors for bioproduction improvement. J. Biosci. Bioeng..

[98] Sharma U.K., Dipankar C. (2010). Transcriptional switching in *Escherichia coli* during stress and starvation by modulation of sigma activity. Fems Microbiol. Rev..

[99] Helmann J.D. (2010). Alternative sigma factors and the regulation of flagellar gene expression. Mol. Microbiol..

[100] Gruber T.M., Bryant D.A. (1997). Molecular systematic studies of eubacteria, using sigma 70-type sigma factors of group 1 and group 2. J. Bacteriol..

[101] Nordlund P.R., Reichard P. (2006). Ribonucleotide reductases. Adv. Enzymol. Relat. Areas Mol. Biol..

[102] Warren M.J., Raux E., Schubert H.L., Escalantesemerena J.C. (2002). The biosynthesis of adenosylcobalamin (vitamin B12). Cheminform.

[103] Traxler M.F., Summers S.M., Nguyen H.T., Zacharia V.M., Conway T. (2010). The global, ppGpp-mediated stringent response to amino acid starvation in *Escherichia coli*. Mol. Microbiol..

[104] Gross M., Marianovsky I., Glaser G. (2010). MazG—A regulator of programmed cell death in *Escherichia coli*. Mol. Microbiol..

[105] Lee S., Kim M.H., Kang B.S., Kim J.S., Kim K.J. (2008). Crystal Structure of *Escherichia coli* MazG, the Regulator of Nutritional Stress Response. J. Biol. Chem..

[106] Clokie M.R.J., Shan J., Bailey S., Jia Y., Krisch H.M., West S., Mann N.H. (2010). Transcription of a ‘photosynthetic’ T4-type phage during infection of a marine cyanobacterium. Environ. Microbiol..

[107] Galperin M.Y., Moroz O.V., Wilson K.S., Murzin A.G. (2006). House cleaning, a part of good housekeeping. Mol. Microbiol..

[108] Eric S.M., Karam J., Dawson M., Trojanowska M., Gauss P., Gold L. (1987). Translational repression: Biological activity of plasmid-encoded bacteriophage T4 RegA protein. J. Mol. Biol..

[109] Adari H.Y., Rose K., Williams K.R., Konigsberg W.H., Spicer E.K. (1985). Cloning, Nucleotide Sequence, and Overexpression of the Bacteriophage T4 regA Gene. Proc. Natl. Acad. Sci. USA.

[110] Karam J., Gold L., Singer B.S., Dawson M. (1981). Translational regulation: Identification of the site on bacteriophage T4 rIIB mRNA recognized by the regA gene function. Proc. Natl. Acad. Sci. USA.

[111] Herrick J., Sclavi B. (2010). Ribonucleotide reductase and the regulation of DNA replication: An old story and an ancient heritage. Mol. Microbiol..

[112] Dwivedi B. (2013). A bioinformatic analysis of ribonucleotide reductase genes in phage genomes and metagenomes. BMC Evol. Biol..

[113] Harrison A.O., Moore R.M., Polson S.W., Wommack K.E. (2019). Reannotation of the Ribonucleotide Reductase in a Cyanophage Reveals Life History Strategies Within the Virioplankton. Front Microbiol..

[114] Gkotsi D.S., Ludewig H., Sharma S.V., Connolly J.A., Dhaliwal J., Wang Y., Unsworth W.P., Taylor R.J.K., Mclachlan M.M.W., Shanahan S. (2019). A marine viral halogenase that iodinates diverse substrates. Nat. Chem..

[115] Gkotsi D.S., Dhaliwal J., Mclachlan M.M., Mulholand K.R., Goss R.J. (2018). Halogenases: Powerful tools for biocatalysis (mechanisms applications and scope). Curr. Opin. Chem. Biol..

[116] Smith D.R.M., Uria A.R., Helfrich E.J.N., Milbredt D., Van Pée K.H., Piel J.R., Goss R.J.M. (2017). An unusual flavin-dependent halogenase from the metagenome of the marine sponge *Theonella swinhoei* WA. ACS Chem. Biol..

[117] Wu J. (2000). Phosphate Depletion in the Western North Atlantic Ocean. Science.

[118] Kazakov A.E., Vassieva O., Gelfand M.S., Osterman A., Overbeek R. (2003). Bioinformatics classification and functional analysis of PhoH homologs. Silico Biol..

[119] Kim S.K., Makino K., Amemura M., Shinagawa H., Nakata A. (1993). Molecular analysis of the phoH gene, belonging to the phosphate regulon in *Escherichia coli*. J. Bacteriol..

[120] Wanner B.L. (1996). Phosphorus assimilation and control of the phosphate regulon. Escherichia coli Salmonella Cell. Mol. Biol..

[121] Ignacio-Espinoza J.C., Sullivan M.B. (2012). Phylogenomics of T4 cyanophages: Lateral gene transfer in the ‘core’ and origins of host genes. Environ. Microbiol..

[122] Weigele P., Raleigh E.A. (2016). Biosynthesis and Function of Modified Bases in Bacteria and Their Viruses. Chem. Rev..

[123] Dragoš A., Andersen A.J., Lozano-Andrade C.N., Kempen P.J., Kovács Á.T., Strube M.L. (2021). Phages carry interbacterial weapons encoded by biosynthetic gene clusters. Curr. Biol..

[124] Waldbauer J.R., Coleman M.L., Rizzo A.I., Campbell K.L., Lotus J., Zhang L. (2019). Nitrogen sourcing during viral infection of marine cyanobacteria. Proc. Natl. Acad. Sci. USA.

[125] Giglione C., Vallon O., Meinnel T. (2003). Control of protein life-span by N-terminal methionine excision. EMBO J..

[126] Van Duin J., Wijnands R. (1981). The function of ribosomal protein S21 in protein synthesis. Eur. J. Biochem..

[127] Mitra A., Nagaraja V. (2012). Under-representation of intrinsic terminators across bacterial genomic islands: Rho as a principal regulator of xenogenic DNA expression. Gene.

[128] Shestakov S., Karbysheva E. (2015). The role of viruses in the evolution of cyanobacteria. Biol. Bull. Rev..

[129] Gasper R., Schwach J., Hartmann J., Holtkamp A., Wiethaus J., Riedel N., Hofmann E., Frankenberg-Dinkel N. (2017). Distinct features of cyanophage-encoded T-type phycobiliprotein lyase ΦCpeT: The role of auxiliary metabolic genes. J. Biol. Chem..

